# Correction: From words to worries: A cross-cultural comparison of parent-child conversations about snakes in early childhood

**DOI:** 10.1371/journal.pone.0358289

**Published:** 2026-09-14

**Authors:** The PLOS One Staff

## Notice of Republication

This article was republished on 28 August 2026, to correct errors in which the order of the authors’ first and surnames were erroneously reversed.

The correct byline should read:

Lori B. Reider, Félix Landry Yuan, Even Y. M. Leung, Karen K. L. Yeung, Samantha Hing Lam Leung,Catherine Wai Ching Hai,Janet Hiu Ching Lei,Vanessa LoBue

The correct citation:

Reider LB, Yuan FL, Leung EYM, Yeung KKL, Leung SHL, Hai CWC, et al. (2026) From words to worries: A cross-cultural comparison of parent-child conversations about snakes in early childhood. PLoS One 21(4): e0347656. https://doi.org/10.1371/journal.pone.0347656

Please download this article again to view the correct version. The originally published, uncorrected article and the republished, corrected articles are provided here for reference.

## Supporting information

S1 File Originally published, uncorrected article. (PDF)

S2 File Republished, corrected article. (PDF)
