# Supplementary material for: From words to worries: A cross-cultural comparison of parent-child conversations about snakes in early childhood
Source: PLoS One. 2026 Apr 29;21(4):e0347656. doi: 10.1371/journal.pone.0347656 (PMC13127902; doi:10.1371/journal.pone.0347656)
Supplement: S1 File — (DOCX) [file pone.0347656.s001.docx]

**Supporting Information**

**S1 Fig. Sample Picture Book Page Layout**

**
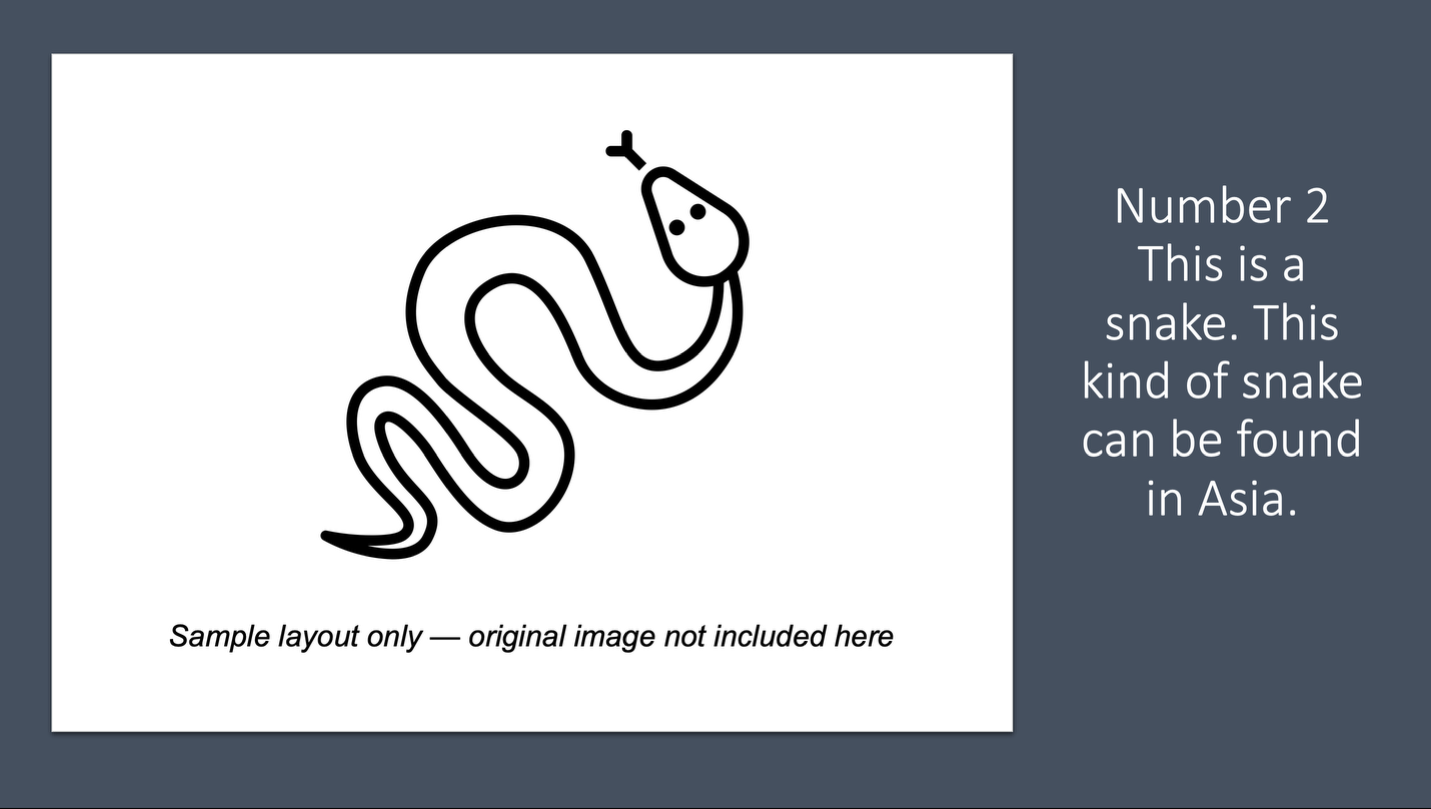
**

*Note.* Sample layout of the pages in the picture book. Original photographic stimuli are not shown due to copyright and the icon is used here to indicate general image placement.

**S1 Table. Children’s Experience with Animals.**

|  | Overall Sample | | HK | | US | |
| --- | --- | --- | --- | --- | --- | --- |
|  | Yes | No | Yes | No | Yes | No |
| Seen snake | 46 | 16 | 20 | 11 | 26 | 5 |
| Seen spider | 53 | 9 | 24 | 7 | 29 | 2 |
| Seen turtle | 59 | 3 | 29 | 2 | 30 | 1 |
| Seen lizard | 50 | 11 | 23 | 8 | 27 | 3 |
| Held/touched snake | 19 | 43 | 8 | 23 | 11 | 20 |
| Held/touched spider | 12 | 50 | 5 | 26 | 7 | 24 |
| Held/touched turtle | 34 | 28 | 17 | 14 | 17 | 14 |
| Held/touched lizard | 20 | 42 | 8 | 23 | 12 | 19 |

*Note.* Values represent the frequency of parents who endorsed each item about their child. No and not sure responses were combined in the table above.

**S2 Table. Children’s Experience with Nature.**

|  | Very often | | | Often | | | Occasionally | | | Never | | | Not sure | | |
| --- | --- | --- | --- | --- | --- | --- | --- | --- | --- | --- | --- | --- | --- | --- | --- |
|  | Overall  n (%) | HK  n (%) | US  n (%) | Overall  n (%) | HK  n (%) | US  n (%) | Overall  n (%) | HK  n (%) | US  n (%) | Overall  n (%) | HK  n (%) | US  n (%) | Overall  n (%) | HK  n (%) | US  n (%) |
| Nature visits | 21 (33.87%) | 3 (9.68%) | 18 (58.06%) | 17 (27.42%) | 12 (38.71%) | 5 (16.13%) | 24 (38.71%) | 16 (51.61%) | 8 (25.81%) | 0 (0%) | 0 (0%) | 0 (0%) | 0 (0%) | 0 (0%) | 0 (0%) |
| Visits the zoo or aquarium | 7 (11.29%) | 1 (3.23%) | 6 (19.35%) | 15 (24.19%) | 7 (22.58%) | 8 (25.81%) | 40 (64.52%) | 23 (74.19%) | 17 (54.84%) | 0 (0%) | 0 (0%) | 0 (0%) | 0 (0%) | 0 (0%) | 0 (0%) |

**S3 Table*.* Descriptives of Parents’ Total Number of Utterances by Valence (Negative, Positive, Neutral), Animal (Snake, Spider, Lizard, Turtle), and Site (Overall Sample, HK, US).**

|  | *Overall Sample* | | *HK* | | *US* | |
| --- | --- | --- | --- | --- | --- | --- |
|  | *Mean* | *SD* | *Mean* | *SD* | *Mean* | *SD* |
| ***Negative*** |  |  |  |  |  |  |
| Snake | 1.79 | 2.61 | 2.39 | 3.23 | 1.17 | 1.58 |
| Spider | 1.80 | 2.20 | 1.57 | 2.13 | 2.03 | 2.29 |
| Lizard | 0.27 | 0.69 | .21 | 0.41 | 0.33 | 0.88 |
| Turtle | 0.17 | 0.42 | 0.17 | 0.46 | 0.17 | 0.38 |
| ***Positive*** |  |  |  |  |  |  |
| Snake | .38 | .80 | .27 | .64 | .50 | .94 |
| Spider | .43 | 1.01 | .37 | .89 | .50 | 1.14 |
| Lizard | .68 | 1.13 | .35 | .55 | 1.00 | 1.44 |
| Turtle | .64 | .80 | .74 | .93 | .53 | .63 |
| ***Neutral*** |  |  |  |  |  |  |
| Snake | 18.51 | 12.26 | 17.70 | 12.70 | 19.29 | 11.98 |
| Spider | 20.33 | 12.18 | 21.17 | 12.35 | 19.52 | 12.15 |
| Lizard | 21.52 | 13.89 | 23.26 | 16.43 | 19.77 | 10.77 |
| Turtle | 20.95 | 12.53 | 20.23 | 13.66 | 21.65 | 11.51 |

**S4 Table**. **Descriptives of Children’s Total Number of Utterances by Valence (Negative, Positive, Neutral), Animal (Snake, Spider, Lizard, Turtle), and Site (Overall Sample, HK, US).**

|  | *Overall Sample* | | *HK* | | *US* | |
| --- | --- | --- | --- | --- | --- | --- |
|  | *Mean* | *SD* | *Mean* | *SD* | *Mean* | *SD* |
| ***Negative*** |  |  |  |  |  |  |
| Snake | 0.75 | 1.18 | 0.63 | 0.93 | 0.87 | 1.38 |
| Spider | 1.27 | 1.56 | 1.03 | 1.28 | 1.52 | 1.79 |
| Lizard | 0.25 | 0.76 | 0.07 | 0.26 | 0.43 | 1.01 |
| Turtle | 0.25 | 0.60 | 0.27 | 0.69 | 0.23 | .50 |
| ***Positive*** |  |  |  |  |  |  |
| Snake | 0.31 | 0.67 | 0.36 | 0.75 | 0.27 | 0.58 |
| Spider | 0.14 | 0.43 | 0.10 | 0.31 | 0.17 | 0.53 |
| Lizard | 0.30 | 0.56 | 0.13 | 0.35 | 0.45 | 0.68 |
| Turtle | 0.25 | 0.54 | 0.20 | 0.48 | 0.30 | 0.60 |
| ***Neutral*** |  |  |  |  |  |  |
| Snake | 11.15 | 7.89 | 10.71 | 9.40 | 11.60 | 6.07 |
| Spider | 12.35 | 8.45 | 12.68 | 8.28 | 12.03 | 8.74 |
| Lizard | 12.90 | 8.29 | 12.81 | 8.70 | 13.00 | 8.00 |
| Turtle | 12.79 | 8.77 | 12.45 | 9.32 | 13.13 | 8.30 |

**S5 Table. Descriptives of Parents’ Proportion of Utterances by Valence (Negative, Positive, Neutral), Animal (Snake, Spider, Lizard, Turtle), and Site (Overall Sample, HK, US).**

|  | *Overall Sample* | | *HK* | | *US* | |
| --- | --- | --- | --- | --- | --- | --- |
|  | *Mean* | *SD* | *Mean* | *SD* | *Mean* | *SD* |
| ***Negative*** |  |  |  |  |  |  |
| Snake | .07 | .09 | .07 | .10 | .06 | .08 |
| Spider | .07 | .10 | .05 | .07 | .10 | .11 |
| Lizard | .01 | .03 | .01 | .03 | .01 | .02 |
| Turtle | .01 | .02 | .01 | .02 | .01 | .02 |
| ***Positive*** |  |  |  |  |  |  |
| Snake | .02 | .03 | .01 | .03 | .02 | .03 |
| Spider | .01 | .03 | .01 | .03 | .02 | .04 |
| Lizard | .02 | .04 | .01 | .02 | .04 | .05 |
| Turtle | .03 | .03 | .03 | .04 | .02 | .03 |
| ***Neutral*** |  |  |  |  |  |  |
| Snake | .91 | .12 | .90 | .13 | .92 | .10 |
| Spider | .91 | .11 | .93 | .08 | .88 | .13 |
| Lizard | .95 | .07 | .96 | .06 | .94 | .07 |
| Turtle | .96 | .05 | .96 | .05 | .96 | .05 |

**S6 Table*.* Descriptives of Children’s Proportion of Utterances by Valence (Negative, Positive, Neutral), Animal (Snake, Spider, Lizard, Turtle), and Site (Overall Sample, HK, US).**

|  | *Overall Sample* | | *HK* | | *US* | |
| --- | --- | --- | --- | --- | --- | --- |
|  | *Mean* | *SD* | *Mean* | *SD* | *Mean* | *SD* |
| ***Negative*** |  |  |  |  |  |  |
| Snake | .05 | .07 | .04 | .06 | .06 | .08 |
| Spider | .09 | .10 | .06 | .08 | .12 | .12 |
| Lizard | .02 | .05 | .01 | .02 | .02 | .06 |
| Turtle | .01 | .02 | .02 | .04 | .01 | .03 |
| ***Positive*** |  |  |  |  |  |  |
| Snake | .02 | .04 | .02 | .04 | .02 | .04 |
| Spider | .01 | .02 | .01 | .02 | .01 | .03 |
| Lizard | .02 | .04 | .01 | .02 | .03 | .05 |
| Turtle | .01 | .03 | .01 | .03 | .01 | .03 |
| ***Neutral*** |  |  |  |  |  |  |
| Snake | .93 | .09 | .94 | .09 | .92 | .10 |
| Spider | .90 | .11 | .93 | .08 | .86 | .12 |
| Lizard | .95 | .08 | .98 | .04 | .93 | .10 |
| Turtle | .96 | .06 | .96 | .05 | .97 | .04 |

**S7 Table. Follow up Tests for 2(Site: US, HK) x 4(Animal: Snake, Spider, Lizard, Turtle).**

*ANOVA on Parents' Negative Language Use (Total Scores)*

| *Main effect of Animal* |  |  |  |
| --- | --- | --- | --- |
| Contrast | estimate | T value | P value |
| Snake - Lizard | 1.48 | 4.30 | <.001** |
| Spider - Lizard | 1.43 | 5.56 | <.001** |
| Snake – Turtle | 1.54 | 4.61 | <.001** |
| Spider - Turtle | 1.49 | 5.73 | <.001** |
| Lizard - Turtle | .06 | .92 | 1.00 |
| Snake – Spider | .05 | .15 | 1.00 |
| *Between-site comparisons for animal* |  |  |  |
| contrast | estimate | T value | P value |
| HK-US: Snake | 1.41 | 2.05 | .04* |
| HK-US: Spider | -.27 | -.50 | .62 |
| HK-US: Lizard | .08 | .67 | .50 |
| HK-US: Turtle | -.03 | -.30 | .76 |
| *Within-site comparisons for each animal* |  |  |  |
| contrast | estimate | T value | P value |
| HK: Lizard - Snake | -2.15 | -4.36 | <.001** |
| HK: Lizard - Spider | -1.26 | -3.42 | .01* |
| HK: Lizard - Turtle | .11 | 1.29 | 1.00 |
| HK: Snake – Spider | .90 | 1.83 | .44 |
| HK: Snake – Turtle | 2.26 | 4.74 | <.001** |
| HK: Spider - Turtle | 1.37 | 3.69 | .003* |
| US: Lizard - Snake | -.82 | -1.70 | .57 |
| US: Lizard - Spider | -1.61 | -4.45 | <.001** |
| US: Lizard - Turtle | .00 | .00 | 1.00 |
| US: Snake – Spider | -.79 | -1.64 | .64 |
| US: Snake – Turtle | .82 | 1.75 | .51 |
| US: Spider - Turtle | 1.61 | 4.41 | <.001** |

*p* < .05*, *p* < .001**; df = 53 for all analyses; Bonferroni method was used to adjust p-values for all follow-up tests.

*Note.* The table represents Bonferroni-adjusted pairwise comparisons for parents’ total negative language use following the significant main effect of animal (Snake, Spider, Lizard, Turtle), and the significant between-subjects effect of site (HK, US) and within-site comparisons of animal.

**S8 Table. Proportion of Utterances: Results for 2(Site: US, HK) x 4(Animal: Snake, Spider, Lizard, Turtle) ANOVA on Parents' Negative Language Use.**

| Predictor | df | *F* | *P* | η^2^ |
| --- | --- | --- | --- | --- |
| Between-Subjects |  |  |  |  |
| Site | 1 | .03 | .87 | <.001 |
| Error | 54 |  |  |  |
|  |  |  |  |  |
| Within-Subjects |  |  |  |  |
| Animal | 3 | 21.40 | <.001** | .28 |
| Site x Animal | 3 | 2.41 | .07 | .04 |
| Error | 162 |  |  |  |

*p* < .05 * *p < .001 ***

*Note.* Results are shown from a 2 (Site: US, HK) × 4 (Animal: Snake, Spider, Lizard, Turtle) ANOVA predicting the proportion of negative utterances by parents. F values, degrees of freedom, p values, and η² are reported.

**S9 Table. Follow up Tests for 2(Site: US, HK) x 4(Animal: Snake, Spider, Lizard, Turtle) ANOVA on Parents' Negative Language Use (Proportion Scores).**

| *Main effect of Animal* |  |  |  |
| --- | --- | --- | --- |
| Contrast | estimate | T value | P value |
| Lizard - Snake | -.06 | -4.87 | <.001** |
| Lizard - Spider | -.07 | -5.44 | <.001** |
| Lizard - Turtle | .001 | .66 | 1.00 |
| Snake – Spider | -.01 | -.63 | 1.00 |
| Snake – Turtle | .06 | 5.09 | <.001** |
| Spider - Turtle | .07 | 5.66 | <.001** |

*p* < .05*, *p* < .001**; df = 54 for all analyses; Bonferroni method was used to adjust p-values for all follow-up tests.

*Note.* The table represents Bonferroni-adjusted pairwise comparisons for parents’ proportion of negative language use following the significant main effect of animal (Snake, Spider, Lizard, Turtle).

**S10 Table**. **Follow up Tests for 2(Site: US, HK) x 4(Animal: Snake, Spider, Lizard, Turtle) ANOVA on Parents' Positive Language Use (Total Scores).**

| *Main effect of Animal* |  |  |  |
| --- | --- | --- | --- |
| Contrast | estimate | T value | P value |
| Lizard - Snake | .30 | 2.21 | .23 |
| Lizard - Spider | .21 | 1.60 | .69 |
| Lizard - Turtle | -.03 | -.22 | 1.00 |
| Snake – Spider | -.09 | -.71 | 1.00 |
| Snake – Turtle | -.33 | -3.03 | .02* |
| Spider - Turtle | -.24 | -1.73 | .53 |

*p* < .05*, *p* < .001**; df = 55 for all analyses; Bonferroni method was used to adjust p-values for all follow-up tests.

*Note.* The table represents Bonferroni-adjusted pairwise comparisons for parents’ total positive language use following the significant main effect of animal (Snake, Spider, Lizard, Turtle).

**S11 Table. Proportion of Utterances: Results for 2(Site: US, HK) x 4(Animal: Snake, spider, lizard, turtle) ANOVA on Parents' Positive Language Use.**

| Predictor | df | *F* | *p* | η^2^ |
| --- | --- | --- | --- | --- |
| Between-Subjects |  |  |  |  |
| Site | 1 | 1.59 | .21 | .03 |
| Error | 54 |  |  |  |
|  |  |  |  |  |
| Within-Subjects |  |  |  |  |
| Animal | 3 | 2.36 | .07 | .04 |
| Site x Animal | 3 | 2.07 | .11 | .04 |
| Error | 162 |  |  |  |

*p* < .05 * *p < .001 ***

*Note.* Results are shown from a 2 (Site: US, HK) × 4 (Animal: Snake, Spider, Lizard, Turtle) ANOVA predicting the proportion of positive utterances by parents. F values, degrees of freedom, p values, and η² are reported.

**S12 Table. Follow up Tests for 2(Site: US, HK) x 4(Animal: Snake, Spider, Lizard, Turtle) ANOVA on Parents' Neutral Language Use (Total Scores).**

| *Main effect of Animal* |  |  |  |
| --- | --- | --- | --- |
| Contrast | estimate | T value | P value |
| Lizard - Snake | 2.36 | 3.23 | .01* |
| Lizard - Spider | .51 | .49 | 1.00 |
| Lizard - Turtle | .09 | .10 | 1.00 |
| Snake – Spider | -1.85 | -1.79 | .48 |
| Snake – Turtle | -2.44 | -2.73 | .05 |
| Spider - Turtle | -.60 | -.59 | 1.00 |

*p* < .05*, *p* < .001**; df = 59 for all analyses; Bonferroni method was used to adjust p-values for all follow-up tests.

*Note.* The table represents Bonferroni-adjusted pairwise comparisons for parents’ total neutral language use following the significant main effect of animal (Snake, Spider, Lizard, Turtle).

**S13 Table. Proportion of Utterances: Results for 2(Site: US, HK) x 4(Animal: Snake, Spider, Lizard, Turtle) ANOVA on Parents' Neutral Language Use.**

| Predictor | df | *F* | *P* | η^2^ |
| --- | --- | --- | --- | --- |
| Between-Subjects |  |  |  |  |
| Site | 1 | .44 | .51 | <.001 |
| Error | 58 |  |  |  |
|  |  |  |  |  |
| Within-Subjects |  |  |  |  |
| Animal | 3 | 7.61 | <.001** | .12 |
| Site x Animal | 3 | 2.07 | .11 | .03 |
| Error | 174 |  |  |  |

*p* < .05 * *p < .001 ***

*Note.* Results are shown from a 2 (Site: US, HK) × 4 (Animal: Snake, Spider, Lizard, Turtle) ANOVA predicting the proportion of neutral utterances by parents. F values, degrees of freedom, p values, and η² are reported.

**S14 Table**. **Follow up Tests for 2(Site: US, HK) x 4(Animal: Snake, Spider, Lizard, Turtle) ANOVA on Parents' Neutral Language Use (Proportion Scores).**

| *Main effect of Animal* |  |  |  |
| --- | --- | --- | --- |
| Contrast | estimate | T value | P value |
| Lizard - Snake | .05 | 3.34 | .01* |
| Lizard - Spider | .04 | 3.04 | .02* |
| Lizard - Turtle | -.01 | -.67 | 1.00 |
| Snake – Spider | -.003 | -.19 | 1.00 |
| Snake – Turtle | -.05 | -3.27 | .01* |
| Spider - Turtle | -.05 | -3.44 | .01* |

*p* < .05*, *p* < .001**; df = 58 for all analyses; Bonferroni method was used to adjust p-values for all follow-up tests.

*Note.* The table represents Bonferroni-adjusted pairwise comparisons for parents’ proportion of neutral language use following the significant main effect of animal (Snake, Spider, Lizard, Turtle).

**S15 Table**. **Follow up Tests for 2(Site: US, HK) x 4(Animal: Snake, Spider, Lizard, Turtle) ANOVA on Children’s Negative Language Use (Total Scores).**

| *Main effect of Animal* |  |  |  |
| --- | --- | --- | --- |
| Contrast | estimate | T value | P value |
| Lizard – Snake | -.40 | 2.31 | .15 |
| Lizard – Spider | -.98 | -4.78 | <.001** |
| Lizard – Turtle | .05 | .40 | 1.00 |
| Snake – Spider | -.58 | -3.15 | .02* |
| Snake – Turtle | .45 | 3.03 | .02* |
| Spider – Turtle | 1.02 | 5.11 | <.001** |

*p* < .05*, *p* < .001**; df = 56 for all analyses; Bonferroni method was used to adjust p-values for all follow-up tests.

*Note.* The table represents Bonferroni-adjusted pairwise comparisons for children’s total negative language use following the significant main effect of animal (Snake, Spider, Lizard, Turtle).

**S16 Table. Proportion of Utterances: Results for 2(Site: US, HK) x 4(Animal: Snake, Spider, Lizard, Turtle) ANOVA on Children's Negative Language Use.**

| Predictor | df | *F* | *P* | η^2^ |
| --- | --- | --- | --- | --- |
| Between-Subjects |  |  |  |  |
| Site | 1 | 3.90 | .05 | .07 |
| Error | 56 |  |  |  |
|  |  |  |  |  |
| Within-Subjects |  |  |  |  |
| Animal | 3 | 18.19 | <.001** | .25 |
| Site x Animal | 3 | 3.92 | .01* | .07 |
| Error | 168 |  |  |  |

*p* < .05 * *p < .001 ***

*Note.* Results are shown from a 2 (Site: US, HK) × 4 (Animal: Snake, Spider, Lizard, Turtle) ANOVA predicting the proportion of negative utterances by children. F values, degrees of freedom, p values, and η² are reported.

**S17 Table. Follow up Tests for 2(Site: US, HK) x 4(Animal: Snake, Spider, Lizard, Turtle) ANOVA on Children’s Negative Language Use (Proportion Scores).**

| *Main effect of Animal* |  |  |  |
| --- | --- | --- | --- |
| Contrast | estimate | T value | P value |
| Lizard – Snake | -.03 | -2.78 | .04* |
| Lizard – Spider | -.07 | -5.10 | <.001** |
| Lizard – Turtle | .001 | .23 | 1.00 |
| Snake – Spider | -.04 | -3.23 | .01 |
| Snake – Turtle | .03 | 3.38 | .01* |
| Spider – Turtle | .08 | 5.67 | <.001** |
| *Between-site comparisons for animal* |  |  |  |
| Contrast | estimate | T value | P value |
| HK-US: Lizard | -.01 | -1.18 | .24 |
| HK-US: Snake | -.01 | -.37 | .71 |
| HK-US: Spider | -.07 | -2.58 | .01* |
| HK-US: Turtle | -.01 | .10 | .33 |
| *Within-site comparisons for each animal* |  |  |  |
| Contrast | estimate | T value | P value |
| HK: Lizard – Snake | -.04 | -2.18 | .20 |
| HK: Lizard – Spider | -.05 | -2.29 | .15 |
| HK: Lizard – Turtle | -.01 | -1.07 | 1.00 |
| HK: Snake – Spider | -.01 | -.65 | 1.00 |
| HK: Snake – Turtle | .03 | 1.80 | .46 |
| HK: Spider – Turtle | .04 | 1.96 | .33 |
| US: Lizard – Snake | -.03 | -1.75 | .52 |
| US: Lizard – Spider | -.10 | -4.97 | <.001** |
| US: Lizard – Turtle | .01 | 1.43 | .95 |
| US: Snake – Spider | -.07 | -3.98 | .001* |
| US: Snake – Turtle | .04 | 3.00 | .02* |
| US: Spider – Turtle | .11 | 6.13 | <.001** |

*p* < .05*, *p* < .001**; df = 56 for all analyses; Bonferroni method was used to adjust p-values for all follow-up tests.

*Note.* The table represents Bonferroni-adjusted pairwise comparisons for children’s proportion of negative language use following the significant main effect of animal (Snake, Spider, Lizard, Turtle), and the significant between-subjects effect of site (HK, US) and within-site comparisons of animal.

**S18 Table. Proportion of Utterances: Results for 2(Site: US, HK) x 4(Animal: Snake, Spider, Lizard, Turtle) ANOVA on Children's Positive Language Use.**

| Predictor | df | *F* | *p* | η^2^ |
| --- | --- | --- | --- | --- |
| Between-Subjects |  |  |  |  |
| Site | 1 | 1.65 | .21 | .03 |
| Error | 53 |  |  |  |
|  |  |  |  |  |
| Within-Subjects |  |  |  |  |
| Animal | 3 | 1.28 | .28 | .02 |
| Site x Animal | 3 | .95 | .42 | .02 |
| Error | 159 |  |  |  |

*p* < .05 * *p < .001 ***

*Note.* Results are shown from a 2 (Site: US, HK) × 4 (Animal: Snake, Spider, Lizard, Turtle) ANOVA predicting the proportion of positive utterances by children. F values, degrees of freedom, p values, and η² are reported.

**S19 Table*.* Proportion of Utterances: Results for 2(Site: US, HK) x 4(Animal: Snake, Spider, Lizard, Turtle) ANOVA on Children's Neutral Language Use.**

| Predictor | df | *F* | *P* | η^2^ |
| --- | --- | --- | --- | --- |
| Between-Subjects |  |  |  |  |
| Site | 1 | 3.02 | .09 | .05 |
| Error | 56 |  |  |  |
|  |  |  |  |  |
| Within-Subjects |  |  |  |  |
| Animal | 3 | 7.11 | <.001** | .11 |
| Site x Animal | 3 | 2.99 | .03* | .03 |
| Error | 168 |  |  |  |

*p* < .05 * *p < .001 ***

*Note.* Results are shown from a 2 (Site: US, HK) × 4 (Animal: Snake, Spider, Lizard, Turtle) ANOVA predicting the proportion of neutral utterances by children. F values, degrees of freedom, p values, and η² are reported.

**S20 Table. Follow up Tests for 2(Site: US, HK) x 4(Animal: Snake, Spider, Lizard, Turtle) ANOVA on Children’s Neutral Language Use (Proportion Scores).**

| *Main effect of Animal* |  |  |  |
| --- | --- | --- | --- |
| Contrast | estimate | T value | P value |
| Lizard – Snake | .02 | 1.74 | .53 |
| Lizard – Spider | .05 | 2.77 | .05 |
| Lizard – Turtle | -.01 | -1.14 | 1.00 |
| Snake – Spider | .03 | 1.77 | .49 |
| Snake – Turtle | -.03 | -2.66 | .06 |
| Spider – Turtle | -.06 | -3.95 | .001* |
| *Between-site comparisons for animal* |  |  |  |
| Contrast | estimate | T value | P value |
| HK-US: Lizard | .05 | 2.15 | .04* |
| HK-US: Snake | .01 | .44 | .66 |
| HK-US: Spider | .06 | 2.20 | .03* |
| HK-US: Turtle | -.02 | -1.06 | .29 |
| *Within-site comparisons for each animal* |  |  |  |
| Contrast | estimate | T value | P value |
| HK: Lizard – Snake | .04 | 2.20 | .19 |
| HK: Lizard – Spider | .04 | 1.70 | .57 |
| HK: Lizard – Turtle | .02 | 1.46 | .91 |
| HK: Snake – Spider | .002 | .10 | 1.00 |
| HK: Snake – Turtle | -.02 | -1.12 | 1.00 |
| HK: Spider – Turtle | -.02 | 1.03 | 1.00 |
| US: Lizard – Snake | .005 | .25 | 1.00 |
| US: Lizard – Spider | .05 | 2.21 | .19 |
| US: Lizard – Turtle | -.04 | -3.06 | .02* |
| US: Snake – Spider | .05 | 2.41 | .12 |
| US: Snake – Turtle | -.05 | -2.64 | .06 |
| US: Spider – Turtle | -.10 | -4.56 | <.001** |

*p* < .05*, *p* < .001**; df = 56 for all analyses; Bonferroni method was used to adjust p-values for all follow-up tests.

*Note.* The table represents Bonferroni-adjusted pairwise comparisons for children’s proportion of neutral language use following the significant main effect of animal (Snake, Spider, Lizard, Turtle), and the significant between-subjects effect of site (HK, US) and within-site comparisons of animal.

**S21 Table. Follow up Tests for 2(Site: US, HK) x 4(Animal: Snake, Spider, Lizard, Turtle) ANOVA on Parents’ Fear of Animals.**

| *Main effect of Site* |  |  |  |
| --- | --- | --- | --- |
| Contrast | estimate | T value | P value |
| HK – US | .37 | 2.79 | .01* |
| *Main effect of Animal* |  |  |  |
| Contrast | estimate | T value | P value |
| Snake – Spider | .58 | 5.85 | <.001** |
| Snake – Lizard | 1.45 | 13.11 | <.001** |
| Snake – Turtle | 2.43 | 19.05 | <.001** |
| Spider – Lizard | .87 | 6.90 | <.001** |
| Spider – Turtle | 1.77 | 14.95 | <.001** |
| Lizard – Turtle | .90 | 9.10 | <.001** |
| *Between-site comparisons for each animal* |  |  |  |
| Contrast | estimate | T value | P value |
| HK-US: Snake | .54 | 2.66 | .01* |
| HK-US: Spider | .32 | 1.43 | .16 |
| HK-US: Lizard | .66 | 3.31 | .001* |
| HK-US: Turtle | -.06 | -.46 | .65 |
| *Within-site comparisons for each animal* |  |  |  |
| Contrast | estimate | T value | P value |
| HK: Snake - Spider | .69 | 4.87 | <.001** |
| HK: Snake - Lizard | 1.39 | 8.76 | <.001** |
| HK: Snake - Turtle | 2.64 | 14.95 | <.001** |
| HK: Spider - Lizard | .70 | 3.86 | .001* |
| HK: Spider - Turtle | 1.95 | 11.50 | <.001** |
| HK: Lizard - Turtle | 1.25 | 8.86 | <.001** |
| US: Snake - Spider | .46 | 3.38 | .01* |
| US: Snake - Lizard | 1.51 | 9.80 | <.001** |
| US: Snake - Turtle | 2.04 | 11.95 | <.001** |
| US: Spider - Lizard | 1.04 | 5.93 | <.001** |
| US: Spider - Turtle | 1.58 | 9.61 | <.001** |
| US: Lizard - Turtle | .54 | 3.93 | .001* |

*p* < .05*, *p* < .001**; df = 58 for all analyses; Bonferroni method was used to adjust p-values for all follow-up tests.

*Note.* The table represents Bonferroni-adjusted pairwise comparisons for parents’ fear scores following the significant main effect of animal (Snake, Spider, Lizard, Turtle), and the significant between-subjects effect of site (HK, US) and within-site comparisons of animal.

**S22 Table. Follow up Tests for 2(Site: US, HK) x 4(Animal: Snake, Spider, Lizard, Turtle) ANOVA on Children’s Fear of Animals.**

| *Main effect of Animal* |  |  |  |
| --- | --- | --- | --- |
| Contrast | estimate | T value | P value |
| Snake – Spider | .05 | .63 | 1.00 |
| Snake – Turtle | .79 | 6.14 | <.001** |
| Snake – Lizard | .78 | 6.13 | <.001** |
| Spider – Turtle | .74 | 5.59 | <.001** |
| Spider – Lizard | .73 | 5.75 | <.001** |
| Lizard – Turtle | .01 | .05 | 1.00 |
| *Between-site comparisons for each animal* |  |  |  |
| Contrast | estimate | T value | P value |
| HK-US: Snake | -.89 | -4.80 | <.001* |
| HK-US: Spider | -1.11 | -6.35 | <.001** |
| HK-US: Turtle | 1.02 | 5.79 | <.001** |
| HK-US: Lizard | .39 | 1.65 | .10 |
| *Within-site comparisons for each animal* |  |  |  |
| Contrast | estimate | T value | P value |
| HK: Snake - Spider | .16 | 1.34 | 1.00 |
| HK: Snake - Lizard | .14 | .78 | 1.00 |
| HK: Snake - Turtle | -.17 | -1.00 | 1.00 |
| HK: Spider - Lizard | -.02 | -.11 | 1.00 |
| HK: Spider - Turtle | -.33 | -1.75 | .51 |
| HK: Lizard - Turtle | .31 | 1.68 | .59 |
| US: Snake - Spider | -.05 | -.46 | 1.00 |
| US: Snake - Lizard | 1.42 | 7.94 | <.001* |
| US: Snake - Turtle | 1.75 | 9.68 | <.001** |
| US: Spider - Lizard | 1.48 | 8.30 | <.001** |
| US: Spider - Turtle | 1.80 | 9.75 | <.001** |
| US: Lizard - Turtle | .32 | 1.78 | .48 |

*p* < .05*, *p* < .001**; df = 59 for all analyses; Bonferroni method was used to adjust p-values for all follow-up tests.

*Note.* The table represents Bonferroni-adjusted pairwise comparisons for children’s fear scores following the significant main effect of animal (Snake, Spider, Lizard, Turtle), and the significant between-subjects effect of site (HK, US) and within-site comparisons of animal.

**S23 Table. Correlations Between Parent and Child Fear.**

|  | Overall Sample | | HK | | US | |
| --- | --- | --- | --- | --- | --- | --- |
| Animal | r | p | r | p | r | p |
| Snake | -.17 | .19 | .15 | .41 | -.03 | .87 |
| Spider | -.12 | .34 | -.03 | .86 | .01 | .96 |
| Turtle | -.01 | .96 | .19 | .30 | -.08 | .67 |
| Lizard | .30 | .02* | .17 | .35 | .32 | .07 |

*p* < .05 *, *p < .001 ***

*Note.* The table represents correlations and statistical significance for parents’ and children’s fear of each animal (Snake, Spider, Lizard, Turtle) for the overall sample and by site (HK, US).

**S24 Table. Correlations Between Parent and Child Use of Negative Language.**

|  | Overall Sample | | HK | | US | |
| --- | --- | --- | --- | --- | --- | --- |
| Animal | r | p | r | p | r | p |
| Snake | 0.55 | <.001** | .65 | <.001** | .66 | <.001** |
| Spider | 0.44 | <.001** | .37 | .04* | .48 | .01* |
| Turtle | 0.69 | <.001** | .88 | <.001** | .61 | <.001** |
| Lizard | 0.69 | <.001** | .28 | .16 | .72 | <.001** |

*p* < .05 *, *p < .001 ***

*Note.* The table represents correlations and statistical significance for parents’ and children’s use of negative language during conversations about each animal (Snake, Spider, Lizard, Turtle) for the overall sample and by site (HK, US).

**S25 Table. Regression: Parents’ Negative Language About Snakes Predicting Children’s Fear of Snakes.**

| **Predictor** | ***b*** | **SE** | **t** | ***p-*value** |
| --- | --- | --- | --- | --- |
| Step 1 |  |  |  |  |
| Constant | 3.27 | .13 | 24.62 | <.001** |
| Negative language | -.01 | .04 | -.15 | .88 |
| Model Fit |  |  |  |  |
| R^2^ | .0004 |  |  |  |
| Adjusted R^2^ | -.02 |  |  |  |
| F statistic | .02 |  |  | .88 |

*p* < .05 *, *p < .001 ***

**S26 Table. Regression: Parents’ Negative Language About Spiders Predicting Children’s Fear of Spiders.**

| **Predictor** | ***b*** | **SE** | **t** | ***p-*value** |
| --- | --- | --- | --- | --- |
| Step 1 |  |  |  |  |
| Constant | 3.09 | .15 | 21.09 | <.001** |
| Negative language | .07 | .05 | 1.45 | .15 |
| Model Fit |  |  |  |  |
| R^2^ | .03 |  |  |  |
| Adjusted R^2^ | .02 |  |  |  |
| F statistic | 2.10 |  |  | .15 |

*p* < .05 *, *p < .001 ***

**S27 Table. Regression: Parents’ Negative Language About Snakes and Site (US, HK) Predicting Children’s Fear of Snakes.**

| **Predictor** | ***b*** | **SE** | **t** | ***p-*value** |
| --- | --- | --- | --- | --- |
| Step 1 |  |  |  |  |
| Constant | 2.72 | .16 | 16.58 | <.001** |
| Negative language | .04 | .04 | 1.03 | .31 |
| Site (US) | .98 | .23 | 4.17 | <.001** |
| Negative language x Site (US) | -.03 | .10 | -.36 | .72 |
| Model Fit |  |  |  |  |
| R^2^ | .29 |  |  |  |
| Adjusted R^2^ | .25 |  |  |  |
| F statistic | 7.81 |  |  | <.001** |

*p* < .05 *, *p < .001 ***

**S28 Table. Regression: Parents’ Negative Language About Spiders and Site (US, HK) Predicting Children’s Fear of Spiders.**

| **Predictor** | ***b*** | **SE** | **t** | ***p-*value** |
| --- | --- | --- | --- | --- |
| Step 1 |  |  |  |  |
| Constant | 2.66 | .16 | 16.60 | <.001** |
| Negative language | .003 | .06 | .05 | .96 |
| Site (US) | .93 | .23 | 4.03 | <.001** |
| Negative language x Site (US) | .09 | .08 | 1.08 | .29 |
| Model Fit |  |  |  |  |
| R^2^ | .43 |  |  |  |
| Adjusted R^2^ | .40 |  |  |  |
| F statistic | 14.01 |  |  | <.001** |

*p* < .05 *, *p < .001 ***

**S29 Table. Correlations Between Children’s Nature Experiences and Children’s Fear.**

|  | Overall Sample | |
| --- | --- | --- |
| Animal | r | p |
| Snake | -.06 | .65 |
| Spider | -.14 | .28 |
| Turtle | .26 | .04* |
| Lizard | .32 | .01* |

*p* < .05 *, *p < .001 ***

*Note.* The table represents correlations and statistical significance for parent report of children’s frequency with nature visits and children’s fear beliefs. The r values represent Spearman’s rank-order correlations.

**S30 Table. Correlations Between Children’s Nature Experiences and Children’ Negative Language Use**

|  | Total Scores | | Proportion Scores | |
| --- | --- | --- | --- | --- |
| Animal | r | p | r | p |
| Snake | -.16 | .22 | -.15 | .25 |
| Spider | .09 | .49 | -.06 | .64 |
| Turtle | -.02 | .89 | .03 | .83 |
| Lizard | .16 | .24 | .19 | .15 |

*p* < .05 *, *p < .001 ***

*Note.* The table represents correlations and statistical significance for parent report of children’s frequency with nature visits and children’s total and proportional use of negative language about each animal. The r values represent Spearman’s rank-order correlations.
