# Supplementary material for: From words to worries: A cross-cultural comparison of parent-child conversations about snakes in early childhood
Source: PLoS One. 2026 Apr 29;21(4):e0347656. doi: 10.1371/journal.pone.0347656 (PMC13127902; doi:10.1371/journal.pone.0347656)
Supplement: Attachment — Submitted filename: PONE-D-25-55348_reviewer.pdf [file pone.0347656.s002.pdf]

PLOS ONE

From Words to Worries: A Cross-Cultural Comparison of Parent-Child Conversations About Snakes and Spiders in Early Childhood

--Manuscript Draft--

Manuscript Number:	PONE-D-25-55348
Article Type:	Research Article
Full Title:	From Words to Worries: A Cross-Cultural Comparison of Parent-Child Conversations About Snakes and Spiders in Early Childhood
Short Title:	A cross-cultural comparison of parent-child conversations about snakes and spiders in early childhood
Corresponding Author:	Lori Beth Reider, Ph.D.<br>Rutgers University Newark<br>Newark, NJ UNITED STATES OF AMERICA
Keywords:	fear learning, cross-cultural fears, parent-child conversations, childhood fears
Abstract:	Fear of animals is shaped by psychological, cultural, and evolutionary influences over time. This study aimed to examine whether conversations about commonly feared animals—and in particular, snakes and spiders—differ among parents and children from different cultures. Parents and children from the United States (n = 31; 28 Mothers, 2 Fathers, 1 Other; 15 Males, 16 Females, Mage = 71.55 months, SDage = 12.56, Rangeage = 42) and Hong Kong (n = 31, 29 Mothers, 2 Fathers; 17 Males, 14 Females, Mage = 76.48 months, SDage = 12.12, Rangeage = 44) read through a picture book of snakes, spiders, lizards, and turtles, and their conversations were coded for emotional content. Children and parents also completed several questionnaires, including a measure of their fear beliefs toward each animal. We found that parents and children across sites were more fearful of and used more negative language about snakes and spiders than lizards and turtles, and parents from Hong Kong expressed more fear and used more negative language about snakes than parents from the United States. However, despite more fear and negative language from Hong Kong parents, children from Hong Kong reported being less fearful of snakes and spiders than children from the United States, while Hong Kong children reported feeling more afraid of turtles and lizards. These findings highlight the prevalence of snake fears and negative information about snakes across different regions of the world, and potential differences in the kinds of input parents convey to children about snakes across cultures.
Order of Authors:	Lori Reider
	Félix Landry Yuan
	Even Y.M. Leung
	Karen K. L. Yeung
	Samantha Hing Lam Leung
	Catherine Wai Ching Hai
	Janet Hiu Ching Lei
	Vanessa LoBue
Additional Information:	
Question	Response
**Financial Disclosure**<br><br>Enter a financial disclosure statement that describes the sources of funding for the work included in this submission. Review the submission guidelines for detailed	Yes

Powered by Editorial Manager® and ProduXion Manager® from Aries Systems Corporation

requirements. View published research articles from *PLOS ONE* for specific examples.<br><br>This statement is required for submission and **will appear in the published article** if the submission is accepted. Please make sure it is accurate.<br><br>**Funded studies**<br>Enter a statement with the following details:<br>• Initials of the authors who received each award<br>• Grant numbers awarded to each author<br>• The full name of each funder<br>• URL of each funder website<br>• Did the sponsors or funders play any role in the study design, data collection and analysis, decision to publish, or preparation of the manuscript?<br><br>Did you receive funding for this work?	
Please add funding details.<br>as follow-up to "**Financial Disclosure**<br><br>Enter a financial disclosure statement that describes the sources of funding for the work included in this submission. Review the submission guidelines for detailed requirements. View published research articles from *PLOS ONE* for specific examples.<br><br>This statement is required for submission and **will appear in the published article** if the submission is accepted. Please make sure it is accurate.	The current research was funded by a grant by the James S. McDonnell Foundation to PI LoBue.

**Funded studies**<br>Enter a statement with the following details:<br>• Initials of the authors who received each award<br>• Grant numbers awarded to each author<br>• The full name of each funder<br>• URL of each funder website<br>• Did the sponsors or funders play any role in the study design, data collection and analysis, decision to publish, or preparation of the manuscript?<br><br>Did you receive funding for this work?"	
Please select the country of your main research funder (please select carefully as in some cases this is used in fee calculation).<br>as follow-up to "**Financial Disclosure**<br><br>Enter a financial disclosure statement that describes the sources of funding for the work included in this submission. Review the submission guidelines for detailed requirements. View published research articles from *PLOS ONE* for specific examples.<br><br>This statement is required for submission and **will appear in the published article** if the submission is accepted. Please make sure it is accurate.<br><br>**Funded studies**<br>Enter a statement with the following details:<br>• Initials of the authors who received each award<br>• Grant numbers awarded to each author<br>• The full name of each funder<br>• URL of each funder website<br>• Did the sponsors or funders play any role in the study design, data collection and analysis, decision to publish, or preparation of the manuscript?<br><br>Did you receive funding for this work?"	UNITED STATES - US
**Competing Interests**	The authors have declared that no competing interests exist.

Use the instructions below to enter a competing interest statement for this submission. On behalf of all authors, disclose any competing interests that could be perceived to bias this work—acknowledging all financial support and any other relevant financial or non-financial competing interests.<br><br>This statement is required for submission and **will appear in the published article** if the submission is accepted. Please make sure it is accurate and that any funding sources listed in your Funding Information later in the submission form are also declared in your Financial Disclosure statement.<br><br>View published research articles from *PLOS ONE* for specific examples.<br><br>**NO authors have competing interests**<br><br>Enter: *The authors have declared that no competing interests exist.*<br><br>**Authors with competing interests**<br><br>Enter competing interest details beginning with this statement:<br><br>*I have read the journal's policy and the authors of this manuscript have the following competing interests: [insert competing interests here]*<br><br>* typeset	
**Ethics Statement**<br><br>Enter an ethics statement for this submission. This statement is required if the study involved:<br><br>• Human participants<br>• Human specimens or tissue<br>• Vertebrate animals or cephalopods<br>• Vertebrate embryos or tissues	All procedures and materials were approved by the Institutional Review Board at Rutgers University (study title: "Learning, Perception, and Belief Revision in Infants, Children, and Adults," #Pro-2020000399) and the Human Research Ethics Committee at the University of Hong Kong (study title: “Learning, Perception, and Belief Revision Towards Snakes in Infants, Children, and Adults,” #EA210495).

• Field research<br><br>Write "N/A" if the submission does not require an ethics statement.<br><br>General guidance is provided below. Consult the submission guidelines for detailed instructions. **Make sure that all information entered here is included in the Methods section of the manuscript.**	

Powered by Editorial Manager® and ProduXion Manager® from Aries Systems Corporation

**Format for specific study types**<br><br>**Human Subject Research (involving human participants and/or tissue)**<br>• Give the name of the institutional review board or ethics committee that approved the study<br>• Include the approval number and/or a statement indicating approval of this research<br>• Indicate the form of consent obtained (written/oral) or the reason that consent was not obtained (e.g. the data were analyzed anonymously)<br><br>**Animal Research (involving vertebrate animals, embryos or tissues)**<br>• Provide the name of the Institutional Animal Care and Use Committee (IACUC) or other relevant ethics board that reviewed the study protocol, and indicate whether they approved this research or granted a formal waiver of ethical approval<br>• Include an approval number if one was obtained<br>• If the study involved *non-human primates*, add *additional details* about animal welfare and steps taken to ameliorate suffering<br>• If anesthesia, euthanasia, or any kind of animal sacrifice is part of the study, include briefly which substances and/or methods were applied<br><br>**Field Research**<br><br>Include the following details if this study involves the collection of plant, animal, or other materials from a natural setting:<br>• Field permit number<br>• Name of the institution or relevant body that granted permission	
**Data Availability**<br><br>Authors are required to make all data underlying the findings described fully available, without restriction, and from the time of publication. PLOS allows rare exceptions to address legal and ethical concerns. See the PLOS Data Policy and FAQ for detailed information.	Yes - all data are fully available without restriction

A Data Availability Statement describing where the data can be found is required at submission. Your answers to this question constitute the Data Availability Statement and **will be published in the article**, if accepted.<br><br>**Important:** Stating 'data available on request from the author' is not sufficient. If your data are only available upon request, select 'No' for the first question and explain your exceptional situation in the text box.<br><br>Do the authors confirm that all data underlying the findings described in their manuscript are fully available without restriction?	
**Describe where the data may be found in full sentences. If you are copying our sample text, replace any instances of XXX with the appropriate details.**<br><br>• If the data are **held or will be held in a public repository**, include URLs, accession numbers or DOIs. If this information will only be available after acceptance, indicate this by ticking the box below. For example: *All XXX files are available from the XXX database (accession number(s) XXX, XXX.*).<br>• If the data are all contained **within the manuscript and/or Supporting Information files**, enter the following: *All relevant data are within the manuscript and its Supporting Information files.*<br>• If neither of these applies but you are able to provide **details of access elsewhere**, with or without limitations, please do so. For example:<br><br>*Data cannot be shared publicly because of [XXX]. Data are available from the XXX Institutional Data Access / Ethics Committee (contact via XXX) for researchers who meet the criteria for access to confidential data.*<br><br>*The data underlying the results presented in the study are available from (include the name of the third party*	The final data, study protocol, and study stimuli can be accessed on Databrary (volume 1857).

and contact information or URL).<br>• This text is appropriate if the data are owned by a third party and authors do not have permission to share the data.<br><br>* typeset	
Additional data availability information:	Tick here if the URLs/accession numbers/DOIs will be available only after acceptance of the manuscript for publication so that we can ensure their inclusion before publication.

Powered by Editorial Manager® and ProduXion Manager® from Aries Systems Corporation

Manuscript

Click here to access/download;Manuscript;Manuscript_submission.docx

From words to worries: a cross-cultural comparison of parent-child conversations about snakes and spiders in early childhood

Lori B. Reider[1*¶], Félix Landry Yuan[2,3*], Even Y.M. Leung[3], Karen K.L. Yeung[4], Samantha Leung Hing Lam[4], Catherine Wai Ching Hai[3], Janet Hiu Ching Lei[5] & Vanessa LoBue[1]

[1] Department of Psychology, Rutgers University, Newark, NJ, US

[2] Institute for Resources, Environment and Sustainability, University of British Columbia, Vancouver, BC, CA

[3]School of Biological Sciences, The University of Hong Kong, Hong Kong SAR, People's Republic of China

[4]Faculty of Science, The University of Hong Kong, Hong Kong SAR, People's Republic of China

[5] Department of Psychiatry, The University of Hong Kong, Hong Kong SAR, People's Republic of China

*indicates joint-first authorship

[¶]Corresponding author

Email: lori.reider@rutgers.edu.

Abstract

Fear of animals is shaped by psychological, cultural, and evolutionary influences over time. This study aimed to examine whether conversations about commonly feared animals—and in particular, snakes and spiders—differ among parents and children from different cultures. Parents and children from the United States (n = 31; 28 Mothers, 2 Fathers, 1 Other; 15 Males, 16 Females, M_{age} = 71.55 months, SD_{age} = 12.56, $Range_{age}$ = 42) and Hong Kong (n = 31, 29 Mothers, 2 Fathers; 17 Males, 14 Females, M_{age} = 76.48 months, SD_{age} = 12.12, $Range_{age}$ = 44) read through a picture book of snakes, spiders, lizards, and turtles, and their conversations were coded for emotional content. Children and parents also completed several questionnaires, including a measure of their fear beliefs toward each animal. We found that parents and children across sites were more fearful of and used more negative language about snakes and spiders than lizards and turtles, and parents from Hong Kong expressed more fear and used more negative language about snakes than parents from the United States. However, despite more fear and negative language from Hong Kong parents, children from Hong Kong reported being less fearful of snakes and spiders than children from the United States, while Hong Kong children reported feeling more afraid of turtles and lizards. These findings highlight the prevalence of snake fears and negative information about snakes across different regions of the world, and potential differences in the kinds of input parents convey to children about snakes across cultures.

Keywords: fear learning, cross-cultural fears, parent-child conversations, childhood fears

Introduction

Animals have played an important role in the lives of humans throughout recorded history, not only as sources of protection, companionship, and food, but also as important symbols in art, religion, and literatur[illegible]ithin these diverse relationships with the natural world, however, not all animals are regarded by humans in the same way. Snakes and spiders, for example, have historically represented evil and fear in many Western cultures, and are the targets of some of the most common fears and phobias throughout the world [1], likely because they represent threats to safety or survival [2]. In fact, snake and spider fears are so common that researchers have suggested they might have an evolutionary origin, endowing humans with a predisposition to not only detect snakes and spiders very quickly in the environment, but to also rapidly acquire a long-lasting fear of these animals on first exposure (see [3] for a review). Indeed, several studies have demonstrated that children, adults, and non-human primates detect snakes and spiders faster than other kinds of animals (e.g., [4-7]) and adults form long-lasting associations between snakes, spiders, and an aversive outcome (e.g., electric shock [3]).

For most modern humans, however, and particularly for humans who live in temperate, industrialized, and urban settings, fear of snakes and spiders is more widespread than the actual threat they pose. Spider-related deaths are extremely rare, and although most spiders are venomous, very few pose a serious health threat to humans [8]. An estimated 1.2 to 5.5 million people a year are envenomed by a snake, of which about 125,000, or about 2% to 10% of these encounters are fatal [9]. Snake bite envenomation is a significant health concern for many marginalized societies in the tropics, especially in agricultural and rural areas [10], warranting its classification as a neglected tropical disease [11]. Asia and Africa, as well as lower income countries, are afflicted by snake bite morbidity and mortality to a higher degree than other

continents or higher income countries [9]. However, while snakebites continue to be a concern throughout the Global South [11], encounters with snakes that lead to injury or death are relatively rare compared to the global population (e.g., 5.5 million envenomations corresponds to 0.068% of the 8.03 billion global population). Thus, while some snake species do constitute a threat to modern-day humans in many parts of the world, their regional significance contrasts with the global pervasiveness of snake fears.

Given the low prevalence of injuries caused by spiders, and the narrower geographical scope of threats by snakes, why are they so widely feared? Fears can be acquired through several pathways, including direct experiences, vicarious observation, the transmission of negative information, or a combination of the three [12, 13]. Given that snake and spider bites are relatively uncommon globally, these fears likely do not result from a direct negative experience for most individuals. However, there are several studies demonstrating that from a young age, children are exposed to a significant amount of negative information about snakes from books, media, and most importantly, from caregivers. In fact, the vast majority (89%) of intense childhood fears reported in the United States and Australia comes from threatening verbal information (e.g., negative information from parents) or seeing something threatening through media [14]. In one study where preschool-aged children walked through a reptile house at a local zoo with a parent, researchers reported that children were more likely to hear negative information about snakes from their parents when compared to other, even perceptually similar animals, like lizards, frogs, and turtles [15]. In a similar study, preschool-aged children were given a simple storybook containing information about where different snakes, spiders, turtles, frogs, and lizards live, and parents were asked to simply read the book with their children. Parents used significantly more negative information about snakes and spiders than turtles, frogs, and lizards, and significantly less positive

information about snakes and spiders than the other animals as well [16]. This suggests that negative information about snakes and spiders is quite common in parent-child interactions in the US and may serve as a source of learning to fear these animals.

To date, most of the studies examining fear-learning of snakes and spiders has come from samples of largely White, middle-class families from Western high-income countries, where life-threatening encounters with snakes and spiders are extremely rare [15-17]. This is important, as the extent to which certain animals are feared can differ substantially across countries and cultures [18]. For example, as we discussed above, the likelihood of negative encounters with snakes in particular is not universal and depends on socio-economic and environmental factors [10, 19]. Accordingly, how snakes are discussed between parents and children, and whether or not fear is transmitted, may depend on how threatening they are in the local environment [20], which itself varies across cultures and ecosystems.

Importantly, diverse cultural practices and traditions centered on snakes exist throughout the world, and especially across Asia, where snake bite incidence and mortality rates are the highest globally [9]. Some are devotional, as in Southwest India, where snake deities are worshiped and snake killings discouraged, often for fear of being cursed and eventually bitten by a snake [21]. Others express utilitarian views, such as in Hong Kong and much of Southern China, where snakes are traditionally consumed as soup for medicinal and nutritional purposes [22, 23], and their skin used in making traditional musical instruments [24, 25]. These various belief systems and traditions contrast with common, non-indigenous American experiences with snakes, which are often limited to captive settings, such as zoos or as pets, as well as child directed books and media [15, 26]. Comparatively, these latter, curated experiences with snakes are gaining popularity in Hong Kong (e.g. [27]), in addition to utilitarian traditions. Overall, both the US and Hong Kong

share complex, and often negative, associations with snakes [28, 29]. In Chinese culture, however, metaphorical references to snakes can be more dualistic, as reflected by the snake's importance and object of awe in the Chinese zodiac and other myths [28, 30]. In all cases, local snake species exist in the environment, but as a result of social, infrastructural, and cultural circumstances, could evoke disparate thoughts and emotions in children.

Varying practices, traditions, and local snake diversity often imply different ways in which people talk about snakes [21, 31-33]. Cultural differences in people's relations with the natural world affect how parents and children speak to each other about animals generally [34](Gelman et al., 2015). These differences can manifest through the words, stories, and meanings ascribed to snakes, whether positive or negative, by parents as they speak with their children [15, 16]. Myths and stories negatively portraying snakes are a potent vehicle for eliciting fear in many cultural or community contexts [33, 35]. However, we still know very little about whether conversations about snakes differ cross-culturally, and whether such conversations lead to changes in children's fear of snakes across cultures where experiences and expectations of snakes differ.

The current study aimed to examine parent-child conversations about snakes and fear of snakes across cultures, with the inclusion of spiders as a commonly feared, yet relatively less medically or culturally salient animal for comparison. We aimed to examine differences among children and caregivers from the United States (US) and Hong Kong (HK), as these regions of the world reflect different cultural attitudes toward snakes which provide a meaningful basis to examine how the cultural context may influence parent-child conversations about snakes and animals more broadly. We asked whether parents and children report greater fear of snakes and spiders than lizards and turtles, and whether this differs by cultural region (US, HK), particularly for snakes. We also asked whether parents and children provide more negative and less positive

information during conversations about snakes and spiders than lizards and turtles, and whether this differs by region. Finally, we explored relations between parents' and children's fear of each animal and parent's use of negative information.

As discussed above, individuals in Asia are afflicted by snake bite morbidity and mortality to a higher degree than other continents or higher income countries like the US [9]. Further, there is evidence that adults from Hong Kong report being more fearful of typically fear-evoking animals when compared to the US [18]. Thus, we predict that Hong Kong parents and children will use more negative language about snakes in particular and express more fear towards snakes than US adults and children. The same might not be true for lizards, turtles, or even spiders. Understanding the cultural differences in how parents and children discuss our most commonly feared animals can help elucidate the role of negative language in children's developing animal fears, and contribute to our understanding of the role of culture and environmental experiences on the development of fear more broadly.

Materials and Methods

The final data, study protocol, and study stimuli can be accessed on Databrary (BLINDED). The design and analysis plans were preregistered on AsPredicted.org (#87741, https://aspredicted.org/7vfh-dzj8.pdf). The original data collection and analysis plan included parent-child dyads from 3 locations: United States (US), Hong Kong (HK), and India, but due to logistical constraints, we were only able to move forward with two sites (US, HK) and therefore all analyses were adjusted accordingly (see analysis plan). All data were collected online during the COVID-19 pandemic, from February 9 through May 26, 2022.

Participants

Participants were recruited for an online study of parent-child interactions during a picture book reading task. Participants were eligible if they lived in the United States or Hong Kong and self-reported their child was between the ages of 4 and 6 years old at the time of sign up for the study. The final sample included 62 parents (57 Mothers, 4 Fathers, 1 Other) and children (32 Males, 30 Females, M_{age} = 74.02 months, SD_{age} = 12.49, $Range_{age}$ = 55), including 31 parent-child dyads from the United States and 31 dyads from Hong Kong. Sample size was based on previous research using similar methodologies with parents and children (e.g., [15, 16]). Sites did not significantly differ by child age, $t(60) = 1.57$, $p = .12$ or by child sex, $\chi^2(1) = .06$, $p = .80$. Below we describe demographic information for each sample separately, as there were slight differences in how questions were asked to align with data collection at each site (e.g., changing the currency for household income).

Hong Kong (HK) Sample

The HK sample included 31 parents (29 Mothers, 2 Fathers) and children (17 Males, 14 Females, M_{age} = 76.48 months, SD_{age} = 12.12, $Range_{age}$ = 44). Parents described their neighborhood as rural (n = 2, 6.45%), suburban (n = 11, 35.48%), or urban (n = 18, 58.06%). Parents self-identified as Ethnic Chinese (n = 31, 100.00%) and identified their children as Ethnic Chinese (n = 31, 100.00%). Parents reported having a bachelor's degree (n = 14, 45.16%), masters or above (n = 10, 32.26%), bachelors and masters or above (n = 1, 3.23%), diploma (non-bachelor) n = 1, 3.23%), diploma (non-bachelor) and bachelors (n = 1, 3.23%), or high school, F4-F.7 (n = 4, 12.90%). Parents also reported their average household income in the last three years of USD $100,000 (HKD $780,000 or more) (n = 14, 45.16%), USD $60,000- $100,000 (HKD $470,000- $780,000) (n = 8, 25.81%), USD $20,000- $40,000 (HKD $160,000- $310,000) (n = 5, 16.13%), USD $40,000- $60,000 (HKD $310,000- $470,000) (n = 3, 9.68%), or USD $20,000 (HKD

$160,000) (n = 1, 3.23%). Children heard Cantonese only (n = 24, 77.42%), Cantonese and another language (n = 3, 9.68%) or Cantonese and two other languages (n = 4, 12.90%). Parents reported their children as having never lived with a pet (n = 18, 58.06%), currently living with a pet (n = 10, 32.26%), or have previously lived with a pet (n = 3, 9.68%). None of the children currently or have ever lived with any of the animals included in the study.

United States (US) Sample

The US sample included 31 parents (28 Mothers, 2 Fathers, 1 Other) and children (15 Males, 16 Females, M_{age} = 71.55 months, SD_{age} = 12.56, $Range_{age}$ = 42). Parents described their neighborhood as rural (n = 3, 9.68%), suburban (n = 22, 70.97%), or urban (n = 6, 19.35%). Parents self-identified as White, not of Hispanic origin (n = 22, 70.97%), Asian/Pacific Islander (n = 5, 16.13%), more than one race/ethnicity (n = 2, 6.45%), American Indian/Alaska Native (n = 1, 3.23%), and one participant did not provide this information (3.23%). Parents identified their children as White, not of Hispanic origin (n = 21, 67.74%), more than one race/ethnicity (n = 5, 16.13%), Asian/Pacific Islander (n = 3, 9.68%), or American Indian/Alaska Native (n = 2, 6.45%). Parents reported having an advanced degree (n = 16, 51.61%), an AA/BA degree (n = 12, 38.71%), a high school diploma/GED (n = 1, 3.23%), some college/trade school (n = 1, 3.23%), or some school no diploma (n = 1, 3.23%). Parents also reported their average household income in the last three years as more than $100,000 per year (n = 16, 51.61%), $40,000-$59,999 (n = 8, 25.81%), $60,000-$100,000 (n = 4, 12.90%), or $20,000-$39,999 (n = 3, 9.68%). Children heard English only (n = 24, 77.42%) or English and another language (n = 7, 22.58%) in the home. Parents reported their children as currently living with a pet (n = 19, 61.29%), having never lived with a pet (n = 6, 19.35%), or having previously lived with a pet (n = 6, 19.35%). Two children were

reported to be currently living with a pet turtle and one child had previously lived with a pet turtle (no other children had ever lived with any of the animals included in the study).

Materials

Picture Book

All study materials were provided to participants using Qualtrics (Qualtrics, Provo, UT) during a live online Zoom session with a researcher. The researcher first provided instructions to parents about how to navigate the book. The picture book contained 16 pages of animals presented in a randomized order, each with a single neutral image of an animal, followed by a brief sentence with the animal category and where the animal lives (e.g., "This is a snake. This kind of snake can be found in Africa."). The book included 4 categories of animals—snakes, spiders, lizards, and turtles—with one animal image per page and 4 different species represented from each animal category. Snakes were selected due to being highly feared animals with different regional cultural associations, spiders were included due to their high fear rates but less prevalent cultural significance, while lizards and turtles are animals that do not commonly elicit fear. These animals have been used in prior research as these animals are commonly found and discussed in similar settings that children are exposed to such as books about animals, trips to the zoo, or other settings (e.g., [15, 16]).

Table 1 includes the 16 specific animals included in the picture book, all of which were used in a previous study [16]. Within each animal category we included 2 species of lower threat and 2 species of higher threat (e.g., venomous) to ensure the affective quality of the stimuli were as balanced as possible. All animal images were found through Google searches or from Zoo websites and included a full body image of each real animal, pictured in isolation and in a neutral position (i.e., not eating or attacking) in their natural habitat.

Table 1. **List of animal species included in the picture book.**

Animal Category	Species	Threat Relevance
Snake 1	Black Mamba	High
Snake 2	King Cobra	High
Snake 3	Garter Snake	Low
Snake 4	Bull Snake	Low
Spider 1	Brown Recluse	High
Spider 2	Funnel Web Spider	High
Spider 3	Jumping Spider	Low
Spider 4	Huntsman Spider	Low
Lizard 1	Gila Monster	High
Lizard 2	Crocodile Monitor	High
Lizard 3	Skink	Low
Lizard 4	Bearded Dragon	Low
Turtle 1	Alligator Snapping Turtle	High
Turtle 2	Matamata turtle	High
Turtle 3	Central American Wood Turtle	Low
Turtle 4	Galapagos Tortoise	Low

Children's Fear Beliefs.

Children completed a modified version of the Fear Beliefs Questionnaire (FBQ; [36]). This questionnaire consisted of 28 questions including seven items (four reverse scored) regarding children's beliefs about each animal category using a 5-point Likert scale presented visually, ranging from 1- *no not at all* to 5- *yes, definitely*. Items were read aloud to the child by the researcher and recorded in Qualtrics during the study session. As was done in previous research

using this measure (e.g., [16]), when children's responses did not match the exact item response (e.g., saying "yes" instead of "yes, probably" or "yes, definitely"), the researcher asked the child to specify between the two options on that side of the scale (e.g., yes probably or yes definitely?), and when a child was unable to make this distinction, the less extreme response was selected (yes, probably or no, not really). A fear beliefs score for each animal category was calculated as the average of the seven items asked about each animal, with higher scores indicating higher fear beliefs.

Parent Knowledge and Fear Belief Survey

Parents completed a survey about their own fear and knowledge of the 16 animals presented in the picture book. For each animal, parents were asked "*Do you know what kind of [animal] this is*?" with the option to respond with "*Yes*" or "*No*". If the parent's response was "*Yes*", they were asked to label the specific animal. As a measure of fear, parents were asked three questions regarding the level of threat, fear, and willingness to approach each animal (reverse scored). For each item, parents indicated their fear beliefs using a 5-point Likert scale, ranging from 1- *no not at all* to 5- *yes, definitely*. A fear beliefs score was calculated by taking the average of all items asked about each animal category (3 items asked for 4 animal species per category, for a total of 12 items per animal category), with higher scores indicating higher fear beliefs.

Child Experiences and Demographics

Parents were asked whether their child had ever seen or held/touched a live snake/spider/lizard/turtle, how often their child took part in nature visits or trips to the zoo or aquarium, and to report on any pet ownership during the child's life. Parents answered additional questions about their child's date of birth, child's biological sex, child's and parent's racial/ethnic

background, parent's relationship to the child, neighborhood type (rural, suburban, urban), languages spoken at home, parent education, household income, and household size.

Procedure

All procedures and materials were approved by the Institutional Review Board at Rutgers University (study title: "Learning, Perception, and Belief Revision in Infants, Children, and Adults," #Pro-2020000399) and the Human Research Ethics Committee at the University of Hong Kong (study title: "Learning, Perception, and Belief Revision Towards Snakes in Infants, Children, and Adults," #EA210495). Participants from the US were recruited through social media, our lab website, the *ChildrenHelpingScience.com* website, and word of mouth. Participants from the HK sample were recruited through word of mouth and through a family-targeted eco-tourism company (Little Woods Nature Education Limited).

The study took place using an online video call using Zoom. Parents and children completed the study from home with a researcher on the other end of the call. Parents provided verbal consent as well as completed an online consent form on behalf of themselves and their child. Parents and children were then asked to read the picture book together as they typically would read a book together. Following the instructions, the researcher turned off their camera and audio, so the picture book was the main activity on the participant's screen. Parents and children then read through the 16 pages of the book in a randomly presented order.

Following the picture book, the child completed the FBQ with the researcher. To begin, the researcher provided the child with instructions, including practicing how to answer questions using the response scale, and then verbally asked each question to each child. Children could respond to questions verbally (e.g., "yes, definitely") or physically (e.g., two thumbs up) using a hand gesture scale with thumbs up and down options, consistent with previous research [16, 37].

Items within each animal category block were presented in a randomized order, and the order of animal categories was also randomized. Following the FBQ, parents then completed the survey about their knowledge and fear of the animals, child experiences, and demographics. After completing the study, participants were debriefed regarding the nature of the study, and any questions were answered prior to the end of the session. In the US sample, families were compensated with a $10.00 Amazon gift card as a thank you for their time and participation. In the HK sample, families were compensated with a certificate of appreciation for their contributions to this study.

Picture Book Conversation Coding

Conversations during the picture book reading were transcribed by individual utterances provided by parents and children for the specific animal page on the screen during the discussion and were checked by a second researcher prior to coding. Conversations were then coded for positive, negative, or neutral information about the animals on each page using a coding scheme used in previous studies [15, 16]. Utterances that were not directly related to the picture book discussion were not coded (e.g., statements about asking for snacks, statements about other animals or content not included in the book). Utterances could be coded as both positive and negative if the utterance referenced both positive and negative information (e.g., “kind of cute, kind of scary, and a little interesting”). Neutral statements were coded anytime information was read directly from the book, or when any questions or statements regarding the book content were provided, but were not emotional (e.g., discussions about what continent the animal lives on). Positive and negative utterances were further coded by the type of emotional language used but were not examined in this analysis.

At each site, a primary coder was trained and coded all the videos. To establish interrater reliability, an additional coder was trained and independently coded 10 of the transcripts (US: n = 31, 32% double coded; HK: n = 31, 32% double coded). Cohen's Kappa (κ) was used to calculate reliability, with values between .60-.79 as moderate agreement, and values above .80 considered as strong agreement between raters [38]. We obtained an average Cohen's κ of .86 (US) and .80 (HK) for positive, negative, and neutral codes, indicating a very good level of agreement. The primary coder's data from each site was used for all analyses. After establishing reliability, the total number of positive, negative, and neutral utterances for each animal category (snakes, spiders, lizards, turtles) were calculated for each speaker (parent, child).

Data Analytic Plan

The original analytic plan was preregistered on AsPredicted.org (#87741, https://aspredicted.org/7vfh-dzj8.pdf). However, because we were unable to collect data from one of the three sites due to logistical constraints, all planned analyses have been adjusted to account for this change (all analyses comparing sites will now compare two sites instead of three sites). Analyses were conducted using R version 4.3.2 [39].

We first describe children's experience with animals and parents' prior knowledge about the animals portrayed in the book. For our main and pre-registered analyses, we first asked whether parents provided different amounts of positive and negative information about snakes and spiders than lizards and turtles across regions. Two separate 2 (site: US, HK) by 4 (animal: snake, spider, lizard, turtle) ANOVAs were conducted to assess differences in positive and negative information provided by parents across sites. We then asked whether children provided different amounts of positive and negative information about snakes and spiders than lizards and turtles across sites. Two separate 2 (site: US, HK) by 4 (animal: snake, spider, lizard, turtle) ANOVAs were conducted

to assess differences in positive and negative information provided by children across sites. We also explored differences in use of neutral language for parents and children using separate 2 (site: US, HK) by 4 (animal: snake, spider, lizard, turtle) ANOVAs. We also explored these same ANOVAs using proportion scores (e.g., proportion of negative, positive, and neutral language used). Next, we asked whether there were differences in parent and child fear beliefs based on the animal category and site. A separate 2 (site: US, HK) by 4 (animal: snake, spider, lizard, turtle) ANOVA was conducted for parents' and children's fear beliefs to examine whether there are differences in fear beliefs toward each animal across the sites. Finally, we explored whether parents' fear correlated with children's fear of the animals, whether parents' use of negative language correlated with children's use of negative language, and whether parents' use of negative language about snakes and spiders predicted children's fear of snakes and spiders. We also explored these same relations by site.

Results

Children's Experience with Animals

In the parent survey administered through Qualtrics, parents were asked to report on their child's experience with animals and nature. First, parents were asked if their child had ever seen or held/touched a live snake, spider, turtle, or lizard snake (Table 2). The majority of children have seen each of the animals (n's = 46 - 59, 74.19% - 95.16%), with fewer children having ever held or touched each of the animals (n's = 19 - 34, 30.65% - 54.84%). Rates of having seen a snake (n = 46) or held/touched a spider (n = 12) were the lowest of all the animals, while rates of having seen (n = 59) or held/touched a turtle (n = 34) were highest of all the animals. There were some site variations in children's experiences with animals. For example, a greater number of children from the US have seen a snake (n = 26), and held or touched a snake (n = 11) than HK children

(seen n = 20, held/touched = 8), with similar patterns for US and HK children having seen a spider (US n = 29, HK n = 24) and having held or touched a spider (US n = 7, HK n = 5).

Table 2. **Experience with animals.**

	Overall Sample		HK		US	
	Yes	No	Yes	No	Yes	No
Seen snake	46	16	20	11	26	5
Seen spider	53	9	24	7	29	2
Seen turtle	59	3	29	2	30	1
Seen lizard	50	11	23	8	27	3
Held/touched snake	19	43	8	23	11	20
Held/touched spider	12	50	5	26	7	24
Held/touched turtle	34	28	17	14	17	14
Held/touched lizard	20	42	8	23	12	19

Note. Values represent the frequency of participants who endorsed each item. No and not sure responses were combined in the table above.

Parents were also asked about how often their child takes part in activities where these animals may be seen (e.g., nature visits, visitors to the zoo or aquarium). All parents reported that their children have taken part in these activities at least occasionally, with more US parents selecting "very often" as the frequency than HK parents (Table 3). There were some site differences with respect to the frequency with which children engage in these activities. For example, more children from the US engaged in nature visits very often (n = 18) and zoo/aquarium visits very often (n = 6) than HK children (nature visits n = 3, zoo/aquarium n = 1).

Table 3. Experience with Nature.

	Very often			Often			Occasionally			Never			Not sure		
	Overall n (%)	HK n (%)	US n (%)	Overall n (%)	HK n (%)	US n (%)	Overall n (%)	HK n (%)	US n (%)	Overall n (%)	HK n (%)	US n (%)	Overall n (%)	HK n (%)	US n (%)
Nature visits	21 (33.87%)	3 (9.68%)	18 (58.06%)	17 (27.42%)	12 (38.71%)	5 (16.13%)	24 (38.71%)	16 (51.61%)	8 (25.81%)	0 (0%)	0 (0%)	0 (0%)	0 (0%)	0 (0%)	0 (0%)
Visits the zoo or aquarium	7 (11.29%)	1 (3.23%)	6 (19.35%)	15 (24.19%)	7 (22.58%)	8 (25.81%)	40 (64.52%)	23 (74.19%)	17 (54.84%)	0 (0%)	0 (0%)	0 (0%)	0 (0%)	0 (0%)	0 (0%)

Parents' Knowledge of Animals

In the Qualtrics survey following the picture book task and child fear beliefs interview, parents were asked to identify each of the animal species presented in the book to assess prior knowledge (Table 4). In general, parents were able to accurately identify certain animal species, including the king cobra snake (n = 43, 69.35%), tortoise (n = 21, 33.87%), snapping turtle (n = 10, 16.13%), bearded dragon (n = 8, 12.90%) and garter snake (n = 7, 11.29%), with six other species accurately identified < 10% of the time and five that were not identified at all. There was some variation in parent's knowledge of animals by site. For example, more US parents (77.42%) accurately labeled the king cobra (the most accurately labeled animal from the book) than HK parents (61.29%). Some US parents also accurately identified the garter snake (22.58%), but no parents accurately identified the black mamba or bull snake. Accuracy rates were low for all spiders presented in the book and at similar rates across sites.

Table 4. Parents' Prior Knowledge of Animals.

		Overall Sample	HK	US
Animal Category	Species	Accurate n (%)	Accurate n (%)	Accurate n (%)
Snake 1	Black Mamba	0 (0%)	0 (0%)	0 (0%)
Snake 2	King Cobra	43 (69.35%)	19 (61.29%)	24 (77.42%)
Snake 3	Garter Snake	7 (11.29%)	0 (0%)	7 (22.58%)
Snake 4	Bull Snake	0 (0%)	0 (0%)	0 (0%)
Spider 1	Brown Recluse	1 (1.61%)	0 (0%)	1 (3.23%)
Spider 2	Funnel Web Spider	0 (0%)	0 (0%)	0 (0%)
Spider 3	Jumping Spider	2 (3.23%)	1 (3.23%)	1 (3.23%)
Spider 4	Huntsman Spider	5 (8.06%)	0 (0%)	5 (16.13%)
Lizard 1	Gila Monster	4 (6.45%)	0 (0%)	4 (12.90%)
Lizard 2	Crocodile Monitor	0 (0%)	0 (0%)	0 (0%)
Lizard 3	Skink	2 (3.23%)	0 (0%)	2 (6.45%)
Lizard 4	Bearded Dragon	9 (14.52%)	0 (0%)	9 (29.03%)
Turtle 1	Alligator Snapping Turtle	10 (16.13%)	2 (6.45%)	8 (25.81%)
Turtle 2	Matamata turtle	0 (0%)	0 (0%)	0 (0%)
Turtle 3	Central American Wood Turtle	1 (1.61%)	0 (0%)	1 (3.23%)
Turtle 4	Galapagos Tortoise	21 (33.87%)	11 (35.48%)	10 (32.26%)

Conversation Analyses

On average, parents produced 99.02 utterances (SD = 68.11, Min = 12, Max = 422) and children produced 60.16 utterances (SD = 39.33, Min = 3, Max = 169) while reading the picture book together. There were no significant differences in the total number of utterances by site for parents, $t(60) = 1.14$, $p = .26$, or children $t(60) = .54$, $p = .59$. For the remaining conversation analyses on total use of positive, negative, and neutral language, we removed 29 data points that were outliers as outlined in our preregistered plan. Outliers were defined across 48 variables as values that exceeded 3 standard deviations from the mean of language type (positive, negative,

neutral) for each speaker (parent and child), site (US and HK), and animal (snakes, spiders, lizards, turtles). This included 6 US parent data points, 7 US child data points, 9 HK parent data points, and 8 HK child data points (only outlier values were dropped—participants remained in the dataset for all other analyses). Following, we also calculated proportion scores, defined as the proportion of positive, negative, and neutral information about each animal and reran analyses examining differences in language by speaker and animal proportion scores. Using the same outlier procedure (defined as more than 3 standard deviations from the mean proportion for each language type by speaker, site, and animal), 39 data points were removed. This included 8 US parent data points, 8 US child data points, 10 HK parent data points, and 13 HK child data points (again, only outlier values were dropped—participants remained in the dataset for all other analyses).

We primarily describe the raw total number of utterances because we believe that use of *any* emotional information may be influential on children's attitudes toward animals, but we also ran analyses with proportion scores to control for the amount that parents and children talked about each animal overall (e.g., a higher proportion of negative information about snakes from parents would indicate the information from parents was more negative about this animal, whereas the raw data will indicate the number of times something negative was referenced independent of the amount they said).

First, we ran separate 2 (site: US, HK) by 4 (animal: snakes, spiders, lizards, turtles) ANOVAs to examine whether parents and children provided different amounts of positive, negative, and neutral information. We ran these analyses for both the total scores and proportion scores. Descriptives are presented in Tables 5-6 (total scores) and Tables 7-8 (proportion scores) for parents' and children's language, respectively. ANOVA results for the total scores are presented in Tables 9a-14a and results for the proportion scores are presented in Tables 9b-14b.

Analyses were conducted using the afex package version 1.4.1 [40] for ANOVAs and the emmeans package version 1.10.6 [41] for post-hoc comparisons.

For parents' total use of negative language (Table 9a, Fig 1), we found a significant main effect of animal, $F(3, 159) = 18.25$, $p < .001$, $\eta^2 = .26$, with parents using significantly more negative language about snakes and spiders than lizards and turtles (p's < .001, S1 Table). No other comparisons were significant at $p < .05$ (S1 Table). We also found a significant site by animal interaction, $F(3, 159) = 3.52$, $p = .02$, $\eta^2 = .06$. To break down the interaction effect, pairwise comparisons were conducted for each site using Bonferroni adjusted p-values (S1 Table). For between-site comparisons for each animal, results indicated that HK parents used significantly more negative language about snakes than US parents ($p = .04$), consistent with our predictions. No other comparisons were significant at $p < .05$. For within-site comparisons for animals (S1 Table), results indicated that Hong Kong parents used significantly more negative language about snakes than turtles ($p < .001$) and lizards ($p < .001$) and used significantly more negative language about spiders than turtles ($p = .003$) and lizards ($p = .01$). Results also indicated that US parents used significantly more negative language about spiders than turtles ($p < .001$) and lizards ($p < .001$). No other comparisons were significant at $p < .05$. When we ran the same analysis using the proportion of negative language, (Table 9b), we found a significant main effect of animal, $F(3, 162) = 21.40$, $p < .001$, $\eta^2 = .28$, with parents using a significantly greater proportion of negative language about snakes and spiders than lizards and turtles (p's < .001). No other comparisons were significant at $p < .05$ (S2 Table).

Fig 1. Results for 2(site: US, HK) x 4(animal: snakes, spiders, lizards, turtles) ANOVA on Parents' Negative Language Use (Total Score Data).

Table 5. Descriptives (Parent Conversation - Total Score Data).

	Overall Sample		*HK*		*US*	
	Mean	*SD*	*Mean*	*SD*	*Mean*	*SD*
Negative						
Snake	1.79	2.61	2.39	3.23	1.17	1.58
Spider	1.80	2.20	1.57	2.13	2.03	2.29
Lizard	0.27	0.69	.21	0.41	0.33	0.88
Turtle	0.17	0.42	0.17	0.46	0.17	0.38
Positive						
Snake	.38	.80	.27	.64	.50	.94
Spider	.43	1.01	.37	.89	.50	1.14
Lizard	.68	1.13	.35	.55	1.00	1.44
Turtle	.64	.80	.74	.93	.53	.63
Neutral						
Snake	18.51	12.26	17.70	12.70	19.29	11.98
Spider	20.33	12.18	21.17	12.35	19.52	12.15
Lizard	21.52	13.89	23.26	16.43	19.77	10.77
Turtle	20.95	12.53	20.23	13.66	21.65	11.51

Table 6. Descriptives (Child Conversation - Total Score Data).

	Overall Sample		*HK*		*US*	
	Mean	*SD*	*Mean*	*SD*	*Mean*	*SD*
Negative						
Snake	0.75	1.18	0.63	0.93	0.87	1.38
Spider	1.27	1.56	1.03	1.28	1.52	1.79
Lizard	0.25	0.76	0.07	0.26	0.43	1.01
Turtle	0.25	0.60	0.27	0.69	0.23	.50
Positive						
Snake	0.31	0.67	0.36	0.75	0.27	0.58
Spider	0.14	0.43	0.10	0.31	0.17	0.53
Lizard	0.30	0.56	0.13	0.35	0.45	0.68
Turtle	0.25	0.54	0.20	0.48	0.30	0.60
Neutral						
Snake	11.15	7.89	10.71	9.40	11.60	6.07
Spider	12.35	8.45	12.68	8.28	12.03	8.74
Lizard	12.90	8.29	12.81	8.70	13.00	8.00

Turtle	12.79	8.77	12.45	9.32	13.13	8.30

Table 7. Descriptives (Parent Conversation - Proportion Score Data).

	Overall Sample		*HK*		*US*	
	Mean	*SD*	*Mean*	*SD*	*Mean*	*SD*
Negative						
Snake	.07	.09	.07	.10	.06	.08
Spider	.07	.10	.05	.07	.10	.11
Lizard	.01	.03	.01	.03	.01	.02
Turtle	.01	.02	.01	.02	.01	.02
Positive						
Snake	.02	.03	.01	.03	.02	.03
Spider	.01	.03	.01	.03	.02	.04
Lizard	.02	.04	.01	.02	.04	.05
Turtle	.03	.03	.03	.04	.02	.03
Neutral						
Snake	.91	.12	.90	.13	.92	.10
Spider	.91	.11	.93	.08	.88	.13
Lizard	.95	.07	.96	.06	.94	.07
Turtle	.96	.05	.96	.05	.96	.05

Table 8. Descriptives (Child Conversation - Proportion Score Data).

	Overall Sample		*HK*		*US*	
	Mean	*SD*	*Mean*	*SD*	*Mean*	*SD*
Negative						
Snake	.05	.07	.04	.06	.06	.08
Spider	.09	.10	.06	.08	.12	.12
Lizard	.02	.05	.01	.02	.02	.06
Turtle	.01	.02	.02	.04	.01	.03
Positive						
Snake	.02	.04	.02	.04	.02	.04
Spider	.01	.02	.01	.02	.01	.03
Lizard	.02	.04	.01	.02	.03	.05
Turtle	.01	.03	.01	.03	.01	.03
Neutral						
Snake	.93	.09	.94	.09	.92	.10
Spider	.90	.11	.93	.08	.86	.12

Lizard	.95	.08	.98	.04	.93	.10
Turtle	.96	.06	.96	.05	.97	.04

Table 9a. Total Scores: Results for 2(site: US, HK) x 4(animal: snakes, spiders, lizards, turtles) ANOVA on Parents' Negative Language Use.

Predictor	df	*F*	*P*	η^2
Between-Subjects				
Site	1	1.18	.28	.02
Error	53			
Within-Subjects				
Animal	3	18.25	<.001**	.26
Site x Animal	3	3.52	.02*	.06
Error	159			

p < .05 * *p < .001* **

Table 9b. Proportion Scores: Results for 2(site: US, HK) x 4(animal: snakes, spiders, lizards, turtles) ANOVA on Parents' Negative Language Use.

Predictor	df	*F*	*P*	η^2
Between-Subjects				
Site	1	.03	.87	<.001
Error	54			
Within-Subjects				
Animal	3	21.40	<.001**	.28
Site x Animal	3	2.41	.07	.04
Error	162			

p < .05 * *p < .001* **

For parents' total use of positive language (Table 10a), we found a significant main effect of animal, $F(3, 165) = 3.01$, $p = .03$, $\eta^2 = .05$, with parents using significantly less positive language about snakes than turtles ($p = .02$). No other comparisons were significant at $p < .05$ (S3 Table).

When we ran the same analysis using the proportion of positive language, nothing was significant at *p* < .05 (Table 10b). For parents total use of neutral language (Table 11a), we found a significant main effect of animal, $F(3, 177) = 2.93$, $p = .04$, $\eta^2 = .05$, with parents using less neutral language about snakes than lizards ($p = .01$). No other comparisons were significant at $p < .05$ (S4 Table). When we ran the same analysis using the proportion of neutral language (Table 11b), we found a significant main effect of animal $F(3, 174) = 7.61$, $p < .001$, $\eta^2 = .12$, with parents using a greater proportion of neutral language about lizards and turtles than snakes (*p*'s < .02) and a greater proportion of neutral language about turtles than spiders (*p's* < .03). No other comparisons were significant at $p < .05$ (S5 Table).

Table 10a. Total Scores: Results for 2(site: US, HK) x 4(animal: snakes, spiders, lizards, turtles) ANOVA on Parents' Positive Language Use.

Predictor	df	*F*	*p*	η^2
Between-Subjects				
Site	1	.83	.37	.01
Error	55			
Within-Subjects				
Animal	3	3.01	.03*	.05
Site x Animal	3	1.57	.20	.03
Error	165			

p < .05 * *p < .001* **

Table 10b. Proportion Scores: Results for 2(site: US, HK) x 4(animal: snakes, spiders, lizards, turtles) ANOVA on Parents' Positive Language Use.

Predictor	df	*F*	*p*	η^2
Between-Subjects				
Site	1	1.59	.21	.03

Error	54			
Within-Subjects				
Animal	3	2.36	.07	.04
Site x Animal	3	2.07	.11	.04
Error	162			

$p < .05$ * $p < .001$ **

Table 11a. Total Scores: Results for 2(site: US, HK) x 4(animal: snakes, spiders, lizards, turtles) ANOVA on Parents' Neutral Language Use.

Predictor	df	*F*	*p*	η^2
Between-Subjects				
Site	1	.00	.95	.00
Error	59			
Within-Subjects				
Animal	3	2.93	.04*	.05
Site x Animal	3	2.21	.09	.04
Error	177			

$p < .05$ * $p < .001$ **

Table 11b. Proportion Scores: Results for 2(site: US, HK) x 4(animal: snakes, spiders, lizards, turtles) ANOVA on Parents' Neutral Language Use.

Predictor	df	*F*	*P*	η^2
Between-Subjects				
Site	1	.44	.51	<.001
Error	58			
Within-Subjects				
Animal	3	7.61	<.001**	.12
Site x Animal	3	2.07	.11	.03
Error	174			

$p < .05$ * $p < .001$ **

For children's total use of negative language (Table 12a), we found a significant main effect of animal, $F(3, 168) = 15.01$, $p < .001$, $\eta^2 = .22$, with children using significantly more negative language about snakes and spiders than turtles (p's < .03), more negative language about spiders than lizards ($p < .001$), and more negative language about spiders than snakes ($p = .02$). No other comparisons were significant at $p < .05$ (S6 Table). When we ran the same analysis using the proportion of negative language, (Table 12b), we found a significant main effect of animal, $F(3, 168) = 18.19$, $p < .001$, $\eta^2 = .25$, with children using a significantly greater proportion of negative language about snakes and spiders than lizards and turtles ($p < .05$). No other comparisons were significant at $p < .05$ (S7 Table). We also found a significant site by animal interaction, $F(3, 168) = 3.92$, $p = .01$, $\eta^2 = .07$. To break down the interaction effect, pairwise comparisons were conducted for each site using Bonferroni adjusted p-values (S7 Table). For between-site comparisons for each animal, results indicated that US children used a significantly greater proportion of negative language about spiders than HK children ($p = .01$), contrary to our predictions. No other comparisons were significant at $p < .05$ (S7 Table). For within-site comparisons for animals (S7 Table), results indicated that US children used a significantly greater proportion of negative language about spiders than lizards, turtles, and snakes ($p < .01$) and a greater proportion of negative language about spiders than turtles ($p < .01$). No other comparisons were significant at $p < .05$, and there were no significant follow-up findings for the HK sample (S7 Table).

Table 12a. Total Scores: Results for 2(site: US, HK) x 4(animal: snakes, spiders, lizards, turtles) ANOVA on Children's Negative Language Use.

Predictor	df	*F*	*P*	η^2
Between-Subjects				

Site	1	2.60	.11	.04
Error	56			
Within-Subjects				
Animal	3	15.01	<.001**	.22
Site x Animal	3	1.18	.32	.02
Error	168			

p < .05 * *p* < *.001* **

Table 12b. Proportion Scores: Results for 2(site: US, HK) x 4(animal: snakes, spiders, lizards, turtles) ANOVA on Children's Negative Language Use.

Predictor	df	*F*	*P*	η^2
Between-Subjects				
Site	1	3.90	.05	.07
Error	56			
Within-Subjects				
Animal	3	18.19	<.001**	.25
Site x Animal	3	3.92	.01*	.07
Error	168			

p < .05 * *p* < *.001* **

There were no significant effects for children's use of positive language (Table 13a) or proportion of positive language (Table 13b) at $p < .05$. There were no significant effects for children's use of neutral language (Table 14a) at p < .05. For children's proportion of neutral language (Table 14b), there was a main effect of animal, $F(3, 168) = 7.11$, p < .001, $\eta^2 = .11$, with children using a significantly greater proportion of neutral language about turtles than spiders ($p < .01$). No other comparisons were significant at $p < .05$ (S8 Table). We also found a significant site by animal interaction, $F(3, 168) = 2.99$, $p = .03$, $\eta^2 = .03$. To break down the interaction effect, pairwise comparisons were conducted for each site using Bonferroni adjusted p-values (S8 Table). For between-site comparisons for each animal, results indicated that HK children used a significantly greater proportion of neutral language about lizards ($p = .04$) and spiders ($p = .03$)

than US children. No other comparisons were significant at $p < .05$ (S8 Table). For within-site comparisons for animals (S8 Table), results indicated that US children used a significantly greater proportion of neutral language about turtles than lizards and spiders (*p's* < .03). No other comparisons were significant at $p < .05$, and there were no significant follow-up findings for the HK sample (S8 Table).

Table 13a. Total Scores: Results for 2(site: US, HK) x 4(animal: snakes, spiders, lizards, turtles) ANOVA on Children's Positive Language Use.

Predictor	df	*F*	*p*	η^2
Between-Subjects				
Site	1	1.17	.28	.02
Error	54			
Within-Subjects				
Animal	3	1.34	.26	.02
Site x Animal	3	1.08	.36	.02
Error	162			

p < .05 * *p* < *.001* **

Table 13b. Proportion Scores: Results for 2(site: US, HK) x 4(animal: snakes, spiders, lizards, turtles) ANOVA on Children's Positive Language Use.

Predictor	df	*F*	*p*	η^2
Between-Subjects				
Site	1	1.65	.21	.03
Error	53			
Within-Subjects				
Animal	3	1.28	.28	.02
Site x Animal	3	.95	.42	.02
Error	159			

p < .05 * *p* < *.001* **

Table 14a. Total Scores: Results for 2(site: US, HK) x 4(animal: snakes, spiders, lizards, turtles) ANOVA on Children's Neutral Language Use.

Predictor	df	*F*	*p*	η^2
Between-Subjects				
Site	1	.00	.96	<.001
Error	59			
Within-Subjects				
Animal	3	2.25	.08	.09
Site x Animal	3	1.25	.29	.02
Error	177			

p < .05 * *p < .001* **

Table 14b. Proportion Scores: Results for 2(site: US, HK) x 4(animal: snakes, spiders, lizards, turtles) ANOVA on Children's Neutral Language Use.

Predictor	df	*F*	*P*	η^2
Between-Subjects				
Site	1	3.02	.09	.05
Error	56			
Within-Subjects				
Animal	3	7.11	<.001**	.11
Site x Animal	3	2.99	.03*	.03
Error	168			

p < .05 * *p < .001* **

Fear Beliefs

Next, a 2 (site: US, Hong Kong) by 4 (animal: snakes, spiders, lizards, turtles) ANOVA was conducted for parents' and children's fear beliefs to examine whether there are differences in fear beliefs toward each animal and whether this differed by site. Analyses were conducted using the afex package version 1.4.1 [40] for ANOVAs and the emmeans package version 1.10.6 [41]

for post-hoc comparisons. For the fear beliefs analyses, we removed 4 data points as outliers, which were data points defined as exceeding 3 standard deviations from the mean of fear beliefs for each speaker (parent and child), site (US and HK) and animal (snakes, spiders, lizards, turtles). This included 2 HK child data points, and 2 HK parent data points. Descriptives for parents' and children's fear of each animal category for the overall sample and by site can be found in Table 15.

Table 15. Descriptives (Fear Beliefs Data).

Speaker	Animal	Overall Sample		HK		US	
		Mean	SD	Mean	SD	Mean	SD
Children	Snakes	3.26	.84	2.82	.41	3.71	.93
	Spiders	3.22	.88	2.66	.33	3.76	.90
	Lizards	2.50	.94	2.71	.51	2.29	1.21
	Turtles	2.48	.86	3.00	.57	1.96	.78
Parents	Snakes	4.00	.83	4.29	.65	3.72	.90
	Spiders	3.40	.91	3.55	.98	3.26	.84
	Lizards	2.55	.90	2.88	.90	2.22	.77
	Turtles	1.64	.48	1.60	.54	1.68	.41

Note. Children's and parents' fear beliefs scores were measured using different measures but the scores and interpretation is the same (higher average scores indicate greater fear beliefs).

For parents' fear of animals (Table 16, Fig 2), we found a significant main effect of site, $F(1, 58) = 7.76$, $p = .01$, $\eta^2 = .12$, with HK parents reporting more fear than US parents overall, $t(58) = 2.79$, $p = .01$. We also found a significant main effect of animal, $F(3, 174) = 164.41$, $p < .001$, $\eta^2 = .74$, with parents reporting significantly greater fear of snakes than spiders, turtles, and

lizards (all *p*'s < .001, S9 Table), significantly greater fear of spiders than turtles and lizards (all *p*'s < .001), and significantly greater fear of lizards than turtles (*p* < .001, S9 Table). We also found a significant site by animal interaction, $F(3, 174) = 3.89$, $p = .01$, $\eta^2 = .06$. To break down the interaction effect, pairwise comparisons were conducted for each site using Bonferroni adjusted p-values (S9 Table). For between-site comparisons for each animal, results indicated that HK parents reported significantly more fear of snakes (*p* = .01) and lizards (*p* = .001) than US parents. For within-site comparisons for animals, results showed similar patterns across sites. HK parents reported significantly more fear of snakes than spiders, lizards, and turtles (all *p*'s < .001), significantly more fear of spiders than lizards and turtles (*p*'s < .01), and significantly greater fear of lizards than turtles (*p* < .001). US parents also reported significantly more fear of snakes than spiders, turtles, and lizards (*p*'s < .01), significantly more fear of spiders than lizards and turtles (*p*'s < .001), and significantly more fear of lizards than turtles (*p* = .001).

Fig 2. Results for 2(site: US, HK) x 4(animal: snakes, spiders, lizards, turtles) ANOVA on Parents' Fear of Animals.

Table 16. Results for 2(site: US, HK) x 4(animal: snakes, spiders, lizards, turtles) ANOVA on Parents' Fear of Animal.

Predictor	df	*F*	*p*	η^2
Between-Subjects				
Site	1	7.76	.01*	.12
Error	58			
Within-Subjects				
Animal	3	164.41	<.001**	.74
Site x Animal	3	3.89	.01*	.06
Error	174			

p < .05 * *p* < *.001* **

For children's fear of animals (Table 17, Figure 3), we found a significant main effect of animal, $F(3, 177) = 25.63$, $p < .001$, $\eta^2 = .30$, with children reporting more fear of snakes and spiders than lizards and turtles (all *p*'s < .001). No other comparisons were significant at $p < .05$ (S10 Table). We also found a significant site by animal interaction, $F(3, 177) = 34.67$, $p < .001$, $\eta^2 = .37$. To break down the interaction effect, pairwise comparisons were conducted for each site using Bonferroni adjusted p-values. For between-site comparisons for each animal, results indicated that US children were more fearful than HK children of snakes ($p < .001$) and spiders ($p < .001$), and HK children were more fearful of turtles than US children ($p < .001$). There were no significant differences for lizards. For within-site comparisons for animals, results indicated that US children were significantly more fearful of snakes and spiders than lizards and turtles (*p*'s < .001). There were no other significant comparisons for the US sample and no significant comparisons for the HK sample at $p < .05$ (S10 Table).

Fig 3. Results for 2(site: US, HK) x 4(animal: snakes, spiders, lizards, turtles) ANOVA on Children's Fear of Animals.

Table 17. Results for 2(site: US, HK) x 4(animal: snakes, spiders, lizards, turtles) ANOVA on Children's Fear of Animals.

Predictor	df	*F*	*p*	η^2
Between-Subjects				
Site	1	1.38	.24	.02
Error	59			

Within-Subjects				
Animal	3	25.63	<.001**	.30
Site x Animal	3	34.67	<.001**	.37
Error	177			

p < .05 * *p* < *.001 ***

Language and Fear

As additional exploratory analyses, we next examined whether parents' fear was correlated with children's fear of each animal category (S11 Table). Across the overall sample, only parents' fear of lizards was significantly and positively correlated with children's fear of lizards (r = .30, p = .01). Parents' fear of snakes, spiders, and turtles were not significantly correlated with children's fear of snakes, spiders, and turtles, respectively, at $p < .05$. We also explored these relations by site, and nothing was significant at $p < .05$ (S11 Table).

We next examined whether parents' use of negative language was correlated with children's use of negative language about each animal (S12 Table). Across the overall sample, parents' use of negative language was positively correlated with children's negative language for each animal (p's < .001). We also explored these relations by site, and the results were similar with the exception that use of negative language about lizards between parents and children from the HK sample was no longer significant at $p < .05$ (S12 Table).

Next, given that parents and children used more negative language during conversations about snakes and spiders and were also more fearful of these animals, we explored whether parents' use of negative language about snakes and spiders predicted children's fear of snakes and spiders. Parents' use of negative language about snakes did not predict children's fear of snakes, $F(1, 59) = .02$, $p = .88$ (S13 Table), and parents' use of negative language about spiders did not predict children's fear of spiders, $F(1, 58) = 2.10$, $p = .15$ (S14 Table). We further explored site

differences by adding site to each model. For snakes, the overall model was significant, $F(3, 57) = 7.81$, $p < .001$ (S15 Table). Site was a significant predictor, in which children in the US reported higher levels of fear than HK children regardless of parents' negative language about snakes (b = 0.98, SE = 0.23, t = 4.17, p < .001). Parents' negative language about snakes nor the interaction between negative language and site were significant at $p < .05$. For spiders, the overall model was significant, $F(3, 56) = 14.01$, $p < .001$ (S16 Table). Site was a significant predictor, in which children in the US reported higher levels of fear than HK children regardless of parents' negative language about spiders (b = 0.93, SE = 0.23, t = 4.03, p < .001). Neither parents' negative language about spiders nor the interaction between negative language and site were significant at $p < .05$.

General Discussion

The current study examined whether parents and children in HK and the US use more negative language during conversations about snakes and spiders than less commonly feared animals like lizards and turtles. We found that parents in both sites used more negative language about snakes and spiders than lizards and turtles, consistent with findings from previous literature [15, 16]. We also found that parents and children from both sites expressed more fear of snakes and spiders than lizards and turtles. However, the most interesting finding here is that while HK parents used significantly more negative language about snakes and reported more fear of snakes than US parents, US children reported being more fearful of snakes than the HK children.

What could explain these seemingly contradictory findings? At least for between-site differences among parents, one factor might be US parents' greater prior knowledge of the animals shown in our study compared to HK parents, with the animals being correctly identified by US parents more often overall. In fact, while the US parents could accurately identify some of the snakes and spiders (2 snakes, 3 lizards, 1 spider and 3 turtles identified by > 1 parent), the HK

parents were nearly at floor (1 snake and 2 turtles identified by > 1 parent). US parents additionally reported that their children experienced nature—both through nature visits and visits to the zoo or aquarium—more often than HK parents. Together this suggests that the US parents might have more experience with or are more knowledgeable about these animals in general. Having less experience or connectedness with nature is known to amplify negative attitudes towards nature and wildlife [42, 43], and fears towards snakes and spiders in particular [44]. In the case of snakes, more informed knowledge about their biology or life history can lead to more positive attitudes [45-48]. Similarly, people with past, curated interactions with snakes tend to display fewer negative attitudes and less fear towards them [49, 50]. Thus the US parents seemingly had more experience with snakes and with nature in general, possibly leading to less fear.

Moreover, felt exposure to snake bite risks could also be driving the higher fear towards and incidence of negative language about snakes in HK parents. Interestingly, the only animal correctly identified by more than half of the HK parents in our study was the highly venomous king cobra, which also happen to be the only animal presented that is native to Hong Kong. In fact, Hong Kong and the surrounding area hosts 47 different snake species, 14 of which are venomous and 8 of which can deliver a fatal bite [51]. A highly urbanized society interspersed with natural landscapes such as forests and mountains, snakes often inhabit areas adjacent to densely populated areas, resulting in conflicts with people [52]. Although again, fatal envenomations are rare, snake bite incidents are common, and continue to be a medical concern in the region [53-56]. While snake species diversity (e.g. [57]) and snake bite incidents [58, 59] are actually higher in some parts of the US, this is mainly limited to southern states. The concern for snake bites could therefore be more prominent in Hong Kong overall given its higher population density, and relatively larger proportion of people living in close proximity to snakes [52].

Overall, while these possibilities support the trends we have observed in parents, our results suggest the opposite in their children. Interestingly, despite the higher incidences of negative language about snakes with HK parents relative to those in the US, we found that US children in our study were more fearful of snakes than HK children. This result contradicts our expectations based on our findings in Hong Kong about higher negative language use in parents and less experience with nature and snakes in children, as well as past work showing adults in Hong Kong to be more fearful of fear-relevant animals than adults in the US [18]. This finding could be the result of cultural differences in emotional expression. Indeed, previous work has demonstrated that negative emotions are less likely to be expressed in children with Chinese than with American cultural orientations [60, 61]. One study found that Hong Kong Chinese middle school children rely less on talking with someone as a regulation strategy for sadness, anger, or fear than their European American counterparts [62]. There is even evidence that this begins in infancy; another study reported that 11-month-old Chinese infants are less facially expressive than European American infants in response to fear-inducing stimuli [63]. Overall, through parent-child conversations, European American parents tend to place a stronger emphasis on the internal, through coaching and teaching their children to express, discuss, and regulate their emotions. Chinese parents, on the other hand, focus less on internally addressing emotions in conversations with their children, and more on external behavior and conduct for bringing an awareness to others' emotions and maintaining group harmony [60, 64]. Future work should therefore look to explore alternative methodologies for assessing snake fears in children that correct for cross-cultural differences in how negative emotions are expressed.

Thus, one of the limitations for this current study is that we assessed snake fears in children through verbal communication, which could have hindered emotional expression in HK

children. We therefore recommend future research cross-culturally assessing animal fears in children to consider non-verbal approaches. In further expanding this research cross-culturally, future studies should also choose a balanced set of animals representative of all geographic regions sampled. In this study, 2 snakes could be found in North America, 1 in Asia, 1 in Africa. Overall, 5 of the 16 animals could be found in North America, while 2 were from Asia. While most parents in our study were able to identify the king cobra, accuracy for other animals was especially low for the Hong Kong sample. The recognition of local snakes in particular varies culturally and can be of significance in the context of venomous species and snakebite risk [65, 66]. Including animals from different continents around the world assesses the recognition of animals commonly featured in media but should be complemented with more locally relevant species to even out rates of parents' knowledge of the animals presented.

To our knowledge, this study is the first to compare parent-child conversations about snakes and spiders between cultures, and the potentially resulting effects on snake fears. Our results suggest that negative language use about and fear towards snakes can be communicated differently from parent to child according to cultural contexts—at least as far as the outward expression of fear is concerned. These findings warrant attention to how parents and educators speak to children about commonly feared animals, and how resulting attitudes towards these animals by children may manifest differently according to place, geography and culture. Conservation outreach and educational efforts addressing children's attitudes towards wildlife, and their fears towards snakes in particular, should take into account the social and natural environments in which they are raised for a culturally-nuanced understanding of the younger generation's relationships with the natural world.

Acknowledgements

We'd like to thank all parents and children who participated and a special thank you to Little Woods Nature Education Limited for their help in the recruitment of participants.

References

1. LoBue V, Adolph KE. Fear in infancy: Lessons from snakes, spiders, heights, and strangers. Developmental Psychology. 2019; 55(9), 1889. https://doi.org/10.1037/dev0000675
2. Öhman A. Of snakes and faces: An evolutionary perspective on the psychology of fear. Scandinavian Journal of Psychology. 2009; 50(6), 543-552. https://doi.org/10.1111/j.1467-9450.2009.00784.x
3. Öhman A, Mineka S. Fears, phobias, and preparedness: toward an evolved module of fear and fear learning. Psychological Review. 2001; 108(3), 483.
4. LoBue V, DeLoache JS. Detecting the snake in the grass: Attention to fear-relevant stimuli by adults and young children. Psychological Science. 2008; 19(3), 284-289. https://doi.org/10.1111/j.1467-9280.2008.02081.x
5. Lobue V, DeLoache JS. What's so special about slithering serpents? Children and adults rapidly detect snakes based on their simple features. Visual Cognition 2011; 19(1), 129-143. https://doi.org/10.1080/13506285.2010.522216

6. Shibasaki M, Kawai N. Rapid detection of snakes by Japanese monkeys (Macaca fuscata): an evolutionarily predisposed visual system. Journal of Comparative Psychology. 2009; 123(2), 131.
7. Kawai, N., & Koda, H. (2016). Japanese monkeys (Macaca fuscata) quickly detect snakes but not spiders: Evolutionary origins of fear-relevant animals. Journal of Comparative Psychology, 130(3), 299. https://doi.org/10.1037/com0000032
8. Gold BS, Dart RC, Barish RA. Bites of venomous snakes. The New England Journal of Medicine. 2002; 347, 347–356. http://dx.doi.org/10.1056/NEJMra013477
9. Afroz A, Siddiquea BN, Chowdhury HA, Jackson, TN, Watt AD. Snakebite envenoming: A systematic review and meta-analysis of global morbidity and mortality. PLoS Neglected Tropical Diseases. 2024; 18(4), e0012080. https://doi.org/10.1371/journal.pntd.0012080
10. Landry Yuan F, Devan-Song A, Yue S, Bonebrake TC. Snakebite Management and One Health in Asia Using an Integrated Historical, Social, And Ecological Framework. The American Journal of Tropical Medicine and Hygiene. 2021; 106(2), 384. https://doi.org/10.4269/ajtmh.21-0848
11. Chippaux JP. Snakebite envenomation turns again into a neglected tropical disease!. Journal of Venomous Animals and Toxins Including Tropical Diseases. 2017; 23, 38. https://doi.org/10.1186/s40409-017-0127-6
12. Rachman S. The conditioning theory of fear acquisition: A critical examination. Behaviour Research and Therapy. 1977; 15(5), 375-387. https://doi.org/10.1016/0005-7967(77)90041-9

13. Rachman S. Neo-conditioning and the classical theory of fear acquisition. Clinical Psychology Review. 1991; 11(2), 155-173. https://doi.org/10.1016/0272-7358(91)90093-A
14. Ollendick TH, King NJ. Origins of childhood fears: An evaluation of Rachman's theory of fear acquisition. Behaviour Research and Therapy. 1991; 29(2), 117-123. https://doi.org/10.1016/0005-7967(91)90039-6
15. Conrad M, Reider LB, LoBue V. Exploring parent–child conversations about live snakes and spiders: Implications for the development of animal fears. Visitor Studies. 2021; 24(1), 58-78. https://doi.org/10.1080/10645578.2020.1865089
16. Reider LB, Mahaffey EM, Barylski B, LoBue V. “It bites!”: The transmission of negative information about snakes and spiders through a naturalistic picture book interaction. Developmental Psychology. 2022; 58(11), 2140. https://doi.org/10.1037/dev0001429
17. Reider LB, LoBue V. The influence of anthropomorphism on children's learning and attitudes toward snakes. Frontiers in Developmental Psychology. 2024; 2, 1356604. https://doi.org/10.3389/fdpys.2024.1356604
18. Davey GC, McDonald AS, Hirisave U, Prabhu GG, Iwawaki S, Ching IJ, et al. A cross-cultural study of animal fears. Behaviour Research and Therapy. 1998; 36(7-8), 735-750. https://doi.org/10.1016/S0005-7967(98)00059-X
19. Kadam P, Ainsworth S, Sirur FM, Patel DC, Kuruvilla JJ, Majumdar DB. Approaches for implementing society-led community interventions to mitigate snakebite envenoming burden: the SHE-India experience. PLoS Neglected Tropical Diseases. 2021; 15(2), e0009078. https://doi.org/10.1371/journal.pntd.0009078

20. Muris P, Field AP. The role of verbal threat information in the development of childhood fear. “Beware the Jabberwock!”. Clinical Child and Family Psychology Review. 2010; 13(2), 129-150. https://doi.org/10.1007/s10567-010-0064-1

21. Landry Yuan F, Ballullaya UP, Roshnath R, Bonebrake TC, Sinu PA. Sacred groves and serpent-gods moderate human–snake relations. People and Nature. 2020; 2(1), 111-122. https://doi.org/10.1002/pan3.10059

22. Wong OF, Hin-Tat F, Shing-Kit-Tommy L, Ka-Keung L, Chak-Wah K, Simpson ID. A preliminary survey of Hong Kong snake shops and the potential snake bite risks for the healthcare system. Transactions of the Royal Society of Tropical Medicine and Hygiene. 2009; 103(9), 931-936. https://doi.org/10.1016/j.trstmh.2009.02.022

23. Landry Yuan F, Yeung CT, Prigge TL, Dufour PC, Sung YH, Dingle C, Bonebrake TC. Conservation and cultural intersections within Hong Kong's snake soup industry. Oryx. 2023; 57(1), 40-47. https://doi.org/10.1017/S0030605321001630

24. Ho WC. The perception of music learning among parents and students in Hong Kong. Bulletin of the Council for Research in Music Education. 2009; (181), 71-93. https://doi.org/10.2307/40319228

25. Qiaoyi Y. Revitalizing Tradition: The Role of the Erhu in Modern Music and Global Culture. International Journal of Education and Humanities. 2024; 4(4), 512-521. https://doi.org/10.58557/(ijeh).v4i4.271

26. Vaughn AK, Larson LR, Peterson MN, Pacifici LB. Factors associated with human tolerance of snakes in the southeastern United States. Frontiers in Conservation Science. 2022; 3, 1016514. https://doi.org/10.3389/fcosc.2022.1016514

27. Chan, SW, Fang WT, LePage B, Wang SM (2024). Assessing the Current Landscape of Reptile Pet Ownership in Hong Kong: A Foundation for Improved Animal Welfare and Future Research Directions. Animals. 2024; 14(12), 1767. https://doi.org/10.3390/ani14121767

28. Lixia W, Eng WB. A Corpus-Based Study On Snake Metaphors In Mandarin Chinese And British English. GEMA Online Journal of Language Studies. 2012; 12(1).

29. Wang F, Zhang M. Different Associations of Animal Words in English and Chinese and the Relevant Translation Strategies. Theory and Practice in Language Studies. 2017; 7(11), 1085-1090. http://dx.doi.org/10.17507/tpls.0711.17

30. Chu MP. Chinese cultural taboos that affect their language & behavior choices. Asian Culture and History. 2009; 1(2), 122.

31. García-López R, Villegas A, Pacheco-Coronel N, Gómez-Álvarez G. Traditional use and perception of snakes by the Nahuas from Cuetzalan del Progreso, Puebla, Mexico. Journal of Ethnobiology and Ethnomedicine. 2017; 13, 1-10. https://doi.org/10.1186/s13002-016-0134-7

32. Babalola O, Jegede HO, Ogunro B. Perceptions, attitudes, and outcomes of human-snake encounters: A retrospective study of an online discussion community in Nigeria. Asian Journal of Ethnobiology. 2022; 3(1). https://doi.org/10.13057/asianjethnobiol/y030101

33. Musah Y, Attuquayefio DK, Pobee AN, Holbech LH. Ophidiophobia, myth generation, and human perceptions: Implications for snake conservation in a typical savanna community of northern Ghana. Human Dimensions of Wildlife. 2022; 27(4), 321-342. https://doi.org/10.1080/10871209.2021.1952357

34. Gelman SA, Mannheim B, Escalante C, Sanchez Tapia I. Teleological talk in parent–child conversations in Quechua. First Language. 2015; 35(4-5), 359-376. https://doi.org/10.1177/0142723715596646

35. Özel M, Prokop P, Uşak M. Cross-cultural comparison of student attitudes toward snakes. Society & Animals. 2009; 17(3), 224-240. https://doi.org/10.1163/156853009X445398

36. Field, A. P., & Lawson, J. (2003). Fear information and the development of fears during childhood: Effects on implicit fear responses and behavioural avoidance. Behaviour Research and Therapy, 41(11), 1277-1293. https://doi.org/10.1016/S0005-7967(03)00034-2

37. Rifkin, L. S., Schofield, C. A., Beard, C., & Armstrong, T. (2016). Adaptation of a paradigm for examining the development of fear beliefs through the verbal information pathway in preschool-age children. Behaviour Research and Therapy, 87, 34-39. https://doi.org/10.1016/j.brat.2016.08.013

38. Landis JR, Koch GG. The measurement of observer agreement for categorical data. Biometrics. 1977:159-74.

39. R Core Team. R: A Language and Environment for Statistical Computing. R Foundation for Statistical Computing, Vienna, Austria. 2023. https://www.R-project.org.

40. Singmann H, Bolker B, Westfall J, Aust F, Ben-Shachar M. afex: Analysis of Factorial Experiments. R package version 1.4-1. 2024. https://CRAN.R-project.org/package=afex.

41. Lenth R. emmeans: Estimated Marginal Means, aka Least-Squares Means. R package version 1.10.6. 2024. https://CRAN.R-project.org/package=emmeans.

42. Soga M, Evans MJ, Yamanoi T, Fukano Y, Tsuchiya K, Koyanagi TF, Kanai T. How can we mitigate against increasing biophobia among children during the extinction of

experience? Biological Conservation. 2020; 242, 108420. https://doi.org/10.1016/j.biocon.2020.108420

43. Soga M, Gaston KJ, Fukano Y, Evans M J. The vicious cycle of biophobia. Trends in Ecology & Evolution. 2023; 38(6), 512-520. DOI: 10.1016/j.tree.2022.12.012

44. Zsido AN, Coelho CM, Polák J. Nature relatedness: A protective factor for snake and spider fears and phobias. People and Nature. 2022; 4(3), 669-682. https://doi.org/10.1002/pan3.10303

45. Kaishauri N, Makashvili M. Correlation between the Knowledge of Snakes and the Snake Fear. Asian Journal for Humanities and Social Studies. 2013; 1(03), 142-145.

46. Alves RRN, Silva VN, Trovão DM, Oliveira JV, Mourão JS, Dias TL, et al. Students' attitudes toward and knowledge about snakes in the semiarid region of Northeastern Brazil. Journal of Ethnobiology and Ethnomedicine. 2014; 10, 1-8. https://doi.org/10.1186/1746-4269-10-30

47. Liordos V, Kontsiotis VJ, Kokoris S, Pimenidou M. The two faces of Janus, or the dual mode of public attitudes towards snakes. Science of the Total Environment. 2018; 621, 670-678. https://doi.org/10.1016/j.scitotenv.2017.11.311

48. Kelly SJ, Kelly JS, Gardner E, Baker J, Monk C, Julian A. Improving attitudes towards adders (Vipera berus) and nature connectedness in primary-age group children. People and Nature. 2023; 5(6), 1908-1921. https://doi.org/10.1002/pan3.10545

49. Ballouard JM, Ajtic R, Balint H, Brito JC, Crnobrnja-Isailovic J, Desmonts D, et al. Schoolchildren and one of the most unpopular animals: Are they ready to protect snakes? Anthrozoös. 2013; 26(1), 93-109. https://doi.org/10.2752/175303713X13534238631560

50. Pinheiro LT, Rodrigues JFM, Borges-Nojosa DM. Formal education, previous interaction and perception influence the attitudes of people toward the conservation of snakes in a large urban center of northeastern Brazil. Journal of Ethnobiology and Ethnomedicine. 2016; 12, 1-8. https://doi.org/10.1186/s13002-016-0096-9

51. AFCD Agriculture Fisheries and Conservation Department. Hong Kong Biodiversity Online. 2023. https://www.afcd.gov.hk/english/conservation/hkbiodiversity. Accessed 23 May 2025.

52. Yue S, Bonebrake TC, Gibson L. Human-snake conflict patterns in a dense urban-forest mosaic landscape. Herpetological Conservation and Biology. 2019; 14(1), 143-154.

53. Fung HT, Lam SK, Lam KK, Kam CW, Simpson ID. A survey of snakebite management knowledge amongst select physicians in Hong Kong and the implications for snakebite training. Wilderness & Environmental Medicine. 2009; 20(4), 364-372. https://doi.org/10.1580/1080-6032-020.004.0364

54. Fung HT. Snakebites in Hong Kong: how to face the possible changes? Hong Kong Journal of Emergency Medicine. 2011; 18(4), 217-220. https://doi.org/10.1177/102490791101800405

55. Hon KLE, Lee KWT, Cheung KL, Ng PC. Big snake, small snake: which wound is worse when bitten? Acta paediatrica. 2009; 98(8), 1363-1365. https://doi.org/10.1111/j.1651-2227.2009.01307.x

56. Mong R, Ng VC, Tse ML. Safety profile of snake antivenom (use) in Hong Kong–a review of 191 cases from 2008 to 2015. Clinical Toxicology. 2017; 55(10), 1066-1071. https://doi.org/10.1080/15563650.2017.1334916

57. Bassett L, Pandelis G. Updated geographic distributions for Texas reptiles. Reptiles & Amphibians. 2024; 31(1), e21492-e21492. https://doi.org/10.17161/randa.v31i1.21492

58. Greene SC, Folt J, Wyatt K, Brandehoff NP. Epidemiology of fatal snakebites in the United States 1989–2018. The American Journal of Emergency Medicine. 2021; 45, 309-316. https://doi.org/10.1016/j.ajem.2020.08.083

59. Ruha AM, Kleinschmidt KC, Greene S, Spyres MB, Brent J, Wax P, et al. The epidemiology, clinical course, and management of snakebites in the North American Snakebite Registry. Journal of Medical Toxicology. 2017; 13, 309-320. https://doi.org/10.1007/s13181-017-0633-5

60. Yang Y, Wang Q. Culture in emotional development. Handbook of Emotional Development. 2019; 569-593.

61. Ip KI, Miller AL, Karasawa M, Hirabayashi H, Kazama M, Wang L, et al. Emotion expression and regulation in three cultures: Chinese, Japanese, and American preschoolers' reactions to disappointment. Journal of Experimental Child Psychology. 2021; 201, 104972. https://doi.org/10.1016/j.jecp.2020.104972

62. Wan KP, Savina E. Emotion regulation strategies in European American and Hong Kong Chinese middle school children. Contemporary School Psychology. 2016; 20, 152-159. https://doi.org/10.1007/s40688-015-0059-5

63. Camras LA, Oster H, Campos J, Campos R, Ujiie T, Miyake K, et al. Production of emotional facial expressions in European American, Japanese, and Chinese infants. Developmental Psychology. 1998; 34(4), 616. https://doi.org/10.1037/0012-1649.34.4.616

64. Doan SN, Wang Q. Maternal discussions of mental states and behaviors: Relations to emotion situation knowledge in European American and immigrant Chinese children.

Child Development. 2010; 81(5), 1490-1503. https://doi.org/10.1111/j.1467-8624.2010.01487.x

65. Bolon I, Durso AM, Botero Mesa S, Ray N, Alcoba G, Chappuis F, et al.. Identifying the snake: First scoping review on practices of communities and healthcare providers confronted with snakebite across the world. PLoS ONE. 2020; 15(3), e0229989. https://doi.org/10.1371/journal.pone.0229989

66. Corbett SW, Anderson B, Nelson B, Bush S, Hayes WK, Cardwell MD. Most lay people can correctly identify indigenous venomous snakes. The American Journal of Emergency Medicine. 2005; 23(6), 759-762. https://doi.org/10.1016/j.ajem.2005.03.008

Author Contributions

Lori B. Reider: Conceptualization, Data curation, Formal analysis, Investigation, Methodology, Visualization, Program administration, Writing - original draft preparation, Writing - review and editing.

Félix Landry Yuan: Conceptualization, Data curation, Investigation, Program administration, Writing - original draft preparation, Writing - review and editing.

Even Y.M. Leung: Data curation, Investigation, Writing – review and editing.

Karen K.L. Yeung: Investigation.

Samantha Leung Hing Lam: Investigation.

Catherine Wai Ching Hai: Investigation.

Janet Hiu Ching Lei: Investigation.

Vanessa LoBue: Conceptualization, Funding acquisition, Methodology, Supervision, Writing - original draft preparation, Writing - review and editing.

Figure1

Click here to access/download;Figure;fig1_parent_neglang.png

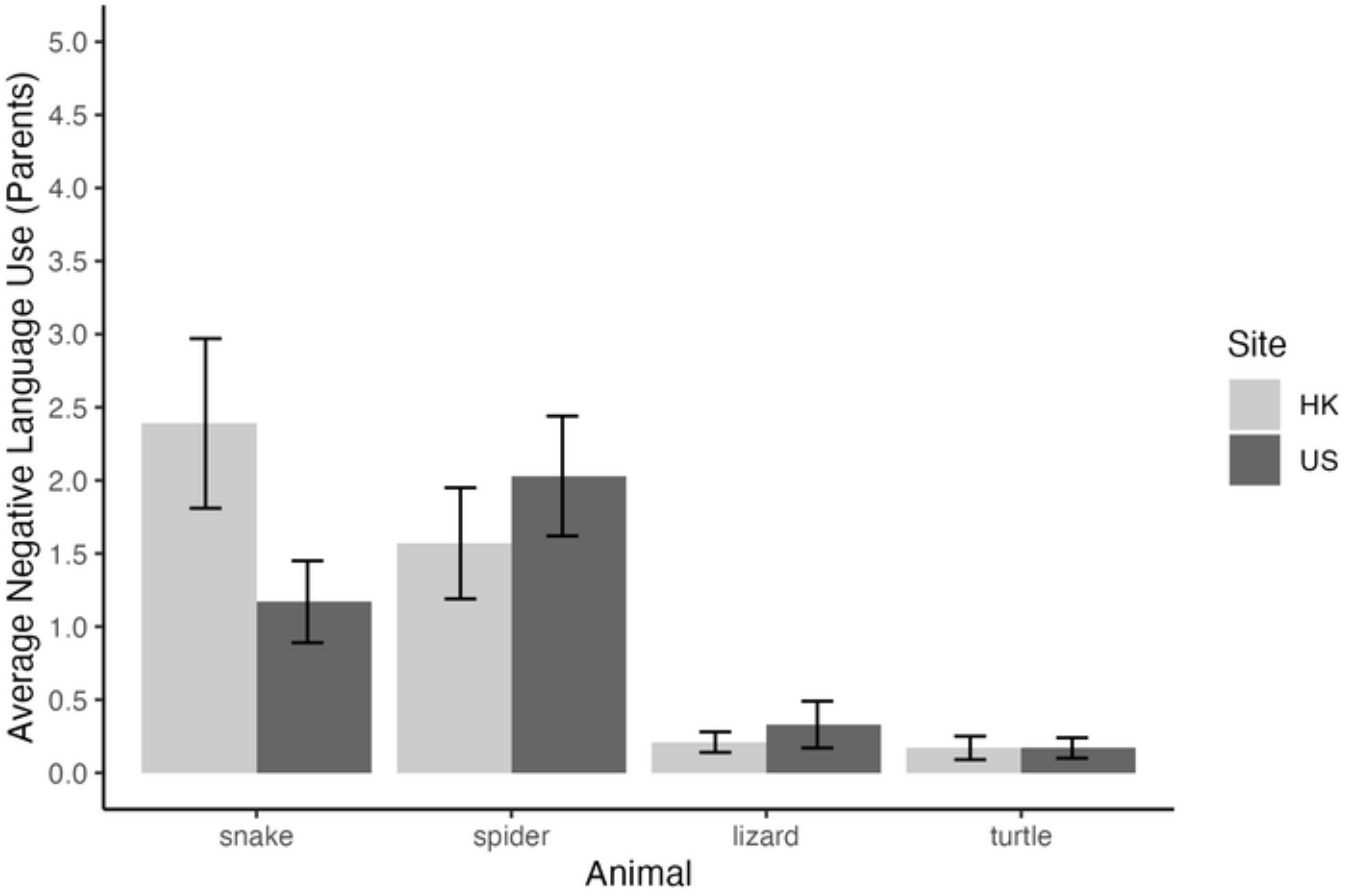

Figure2

Click here to access/download;Figure;fig2_p_fear_plot.png

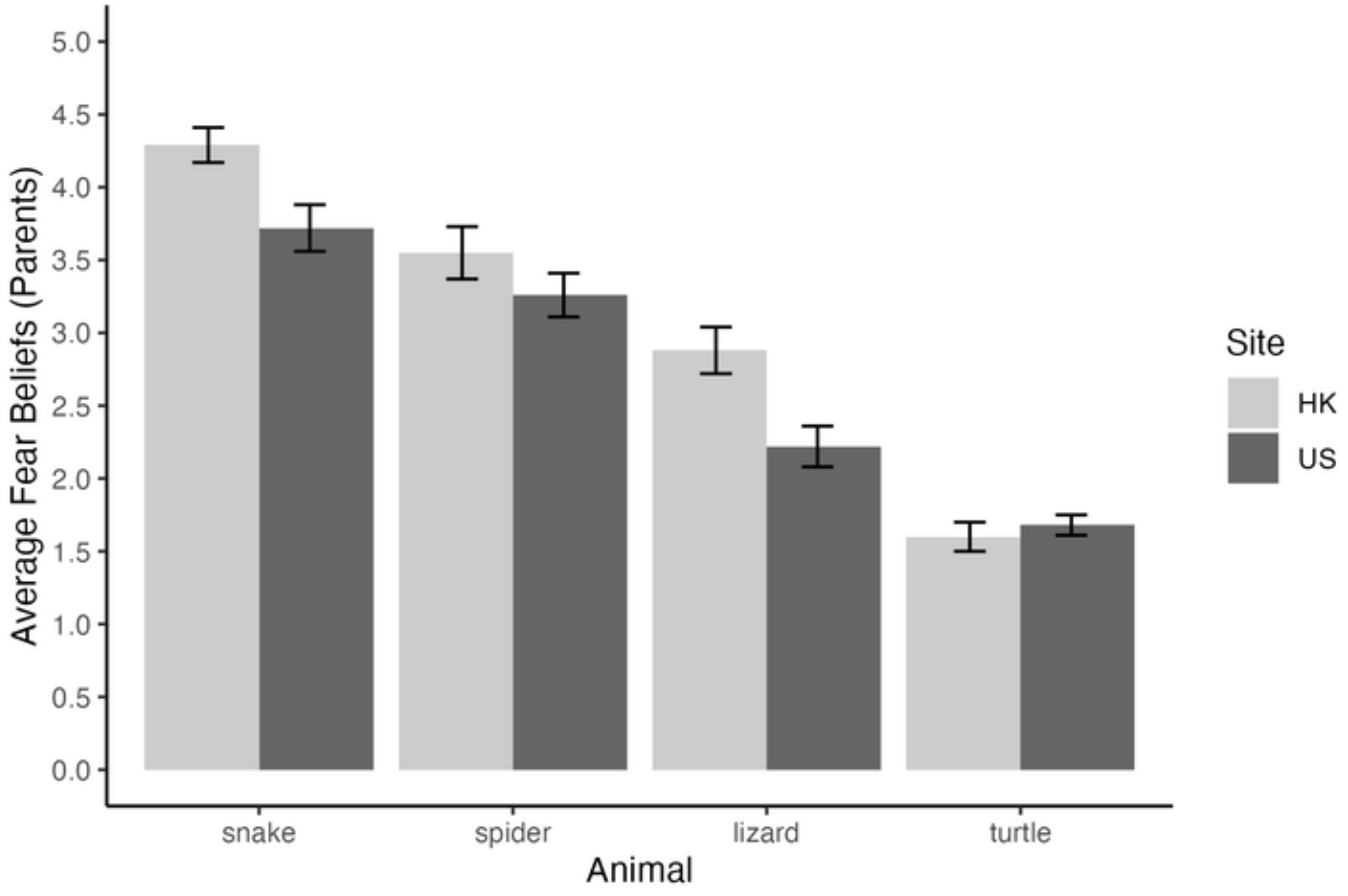

Figure3

Click here to access/download;Figure;fig3_c_fear_plot.png

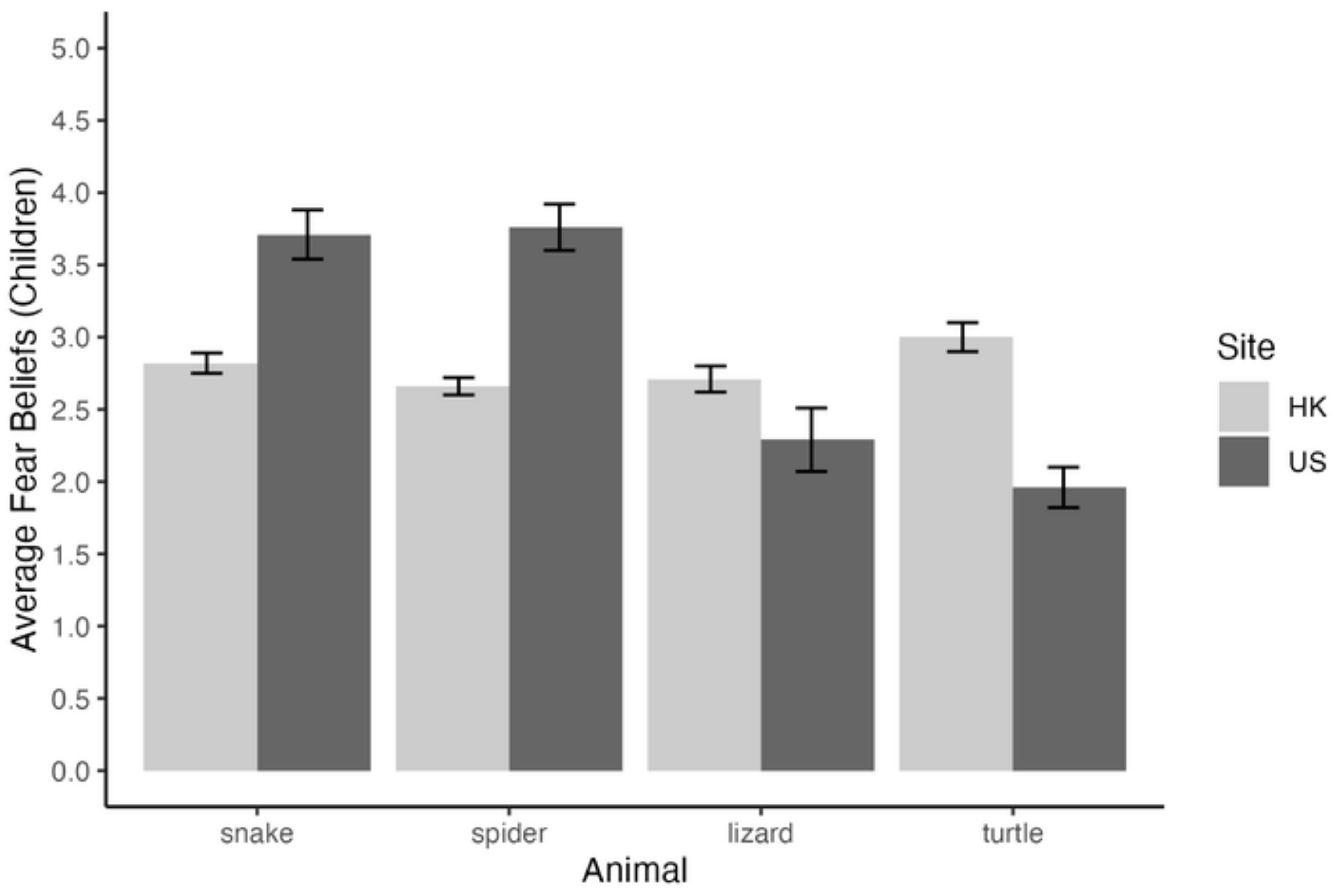

Click here to access/download
Supporting Information
Supporting Information.docx

Click here to access/download
Supporting Information - Compressed/ZIP File Archive
Human Subjects Checklist.zip
